# Supplementary material for: Moving beyond bimetallic-alloy to single-atom dimer atomic-interface for all-pH hydrogen evolution
Source: Nat Commun. 2021 Nov 19;12:6766. doi: 10.1038/s41467-021-27145-3 (PMC8604929; doi:10.1038/s41467-021-27145-3)
Supplement: Supplementary file 1 — Supplementary Information [file 41467_2021_27145_MOESM1_ESM.pdf]

Supplementary Information

Moving Beyond Bimetallic-Alloy to Single-Atom Dimer Atomic-Interface for All-pH Hydrogen Evolution

Ashwani Kumar[1,2,9], Viet Q. Bui[1,2,9], Jinsun Lee[1,2], Lingling Wang[1,2], Amol R. Jadhav[1], Xinghui Liu[1,2], Xiaodong Shao[1,2], Yang Liu[1,2], Jianmin Yu[1,2], Yosep Hwang[1,2], Huong T. D. Bui[1,2], Sara Ajmal[1,2], Min Gyu Kim[3], Seong-Gon Kim[4], Gyeong-Su Park[5], Yoshiyuki Kawazoe[6], and Hyoyoung Lee[* 1,2,7,8]

[1]Center for Integrated Nanostructure Physics (CINAP), Institute for Basic Science (IBS), Sungkyunkwan University, Suwon 16419, Korea.

[2]Department of Chemistry, Sungkyunkwan University (SKKU), Suwon, 16419, Republic of Korea.

[3]Beamline Research Division, Pohang Accelerator Laboratory (PAL), Pohang University of Science and Technology, Pohang 37673, Republic of Korea.

[4]Department of Physics & Astronomy and Center for Computational Sciences, Mississippi State University, Mississippi State, MS 39762, USA.

[5]Department of Materials Science and Engineering and Research Institute of Advanced Materials, Seoul National University, Seoul 08826, Republic of Korea.

[6]New Industry Creation Hatchery Center, Tohoku University, Sendai, 980-8579, Japan.

[7]Department of Biophysics, Sungkyunkwan University, Suwon, 16419, Republic of Korea.

[8]Creative Research Institute, Sungkyunkwan University, Suwon, 16419, Republic of Korea.

[9]These authors contributed equally: Ashwani Kumar, Viet Q. Bui.

*Email of correspondence: hyoyoung@skku.edu

Supplementary Table 1. **Formation energy, averaged Mulliken charge difference (Δq (e^-): Δq is computed as the difference of Mulliken charges on the metal SAD centers), d-band center of the metal atoms, and the bond distance between two metal atoms in TM-SAD-N_6C**.

	CoCu	NiCo	CoFe	CoMn	CuCu	NiNi	CoCo	FeFe	MnMn
**Formation energy (eV)**	- 7.04	-8.86	-10.63	-11.25	-4.24	-7.99	-9.75	-10.82	-11.93
**Average Mulliken charges (e^-)**	0.91	0.88	0.91	0.98	0.99	0.80	0.86	0.89	0.9
**d-band (eV)**	-1.67	-0.87	-1.07	-0.97	-2.82	-0.17	-1.50	-1.05	-0.89
**d_{M-M} (Å)**	2.41	2.32	2.17	2.28	2.40	2.36	2.24	2.11	1.78

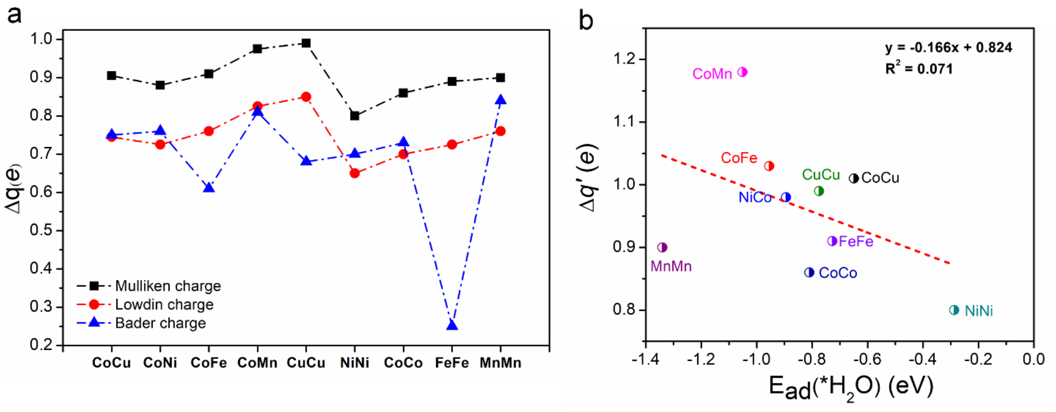

Supplementary Figure 1 | Calculated charge transfers and correlation with the water adsorption energy. **a,** Average Mulliken (Δq (e^-)), Lowdin and Bader charges of TM-SAD center. **b**, Calculated correlation between water adsorption energy and charge transfer from metal-active atom in the TM-SAD-N_6C. Δq’ (e^-) is computed as the difference of Mulliken charges on the metal atom at the active site when isolated and when on the support (SAD-N_6C), with a positive value indicating electron withdrawal from active-metal atom.

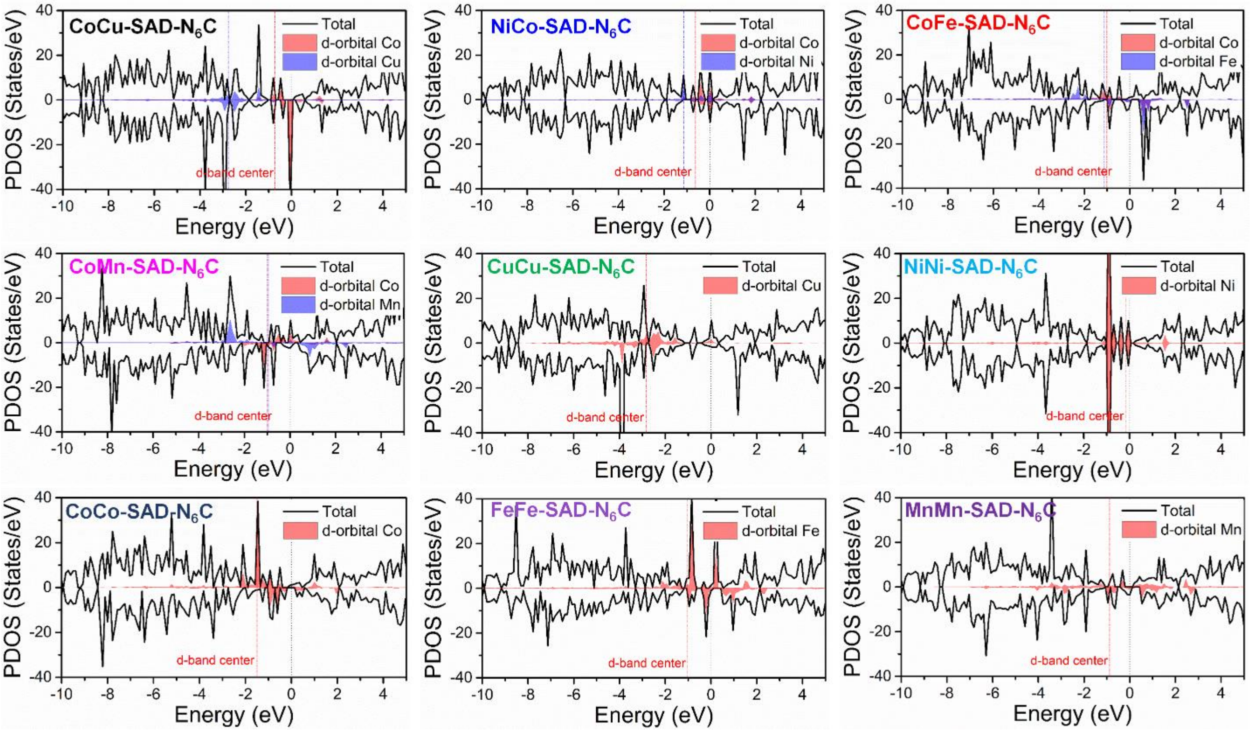

Supplementary Figure 2 | The projected density of states of total and d-orbitals of the metal atoms in TM-SAD-N_6C.

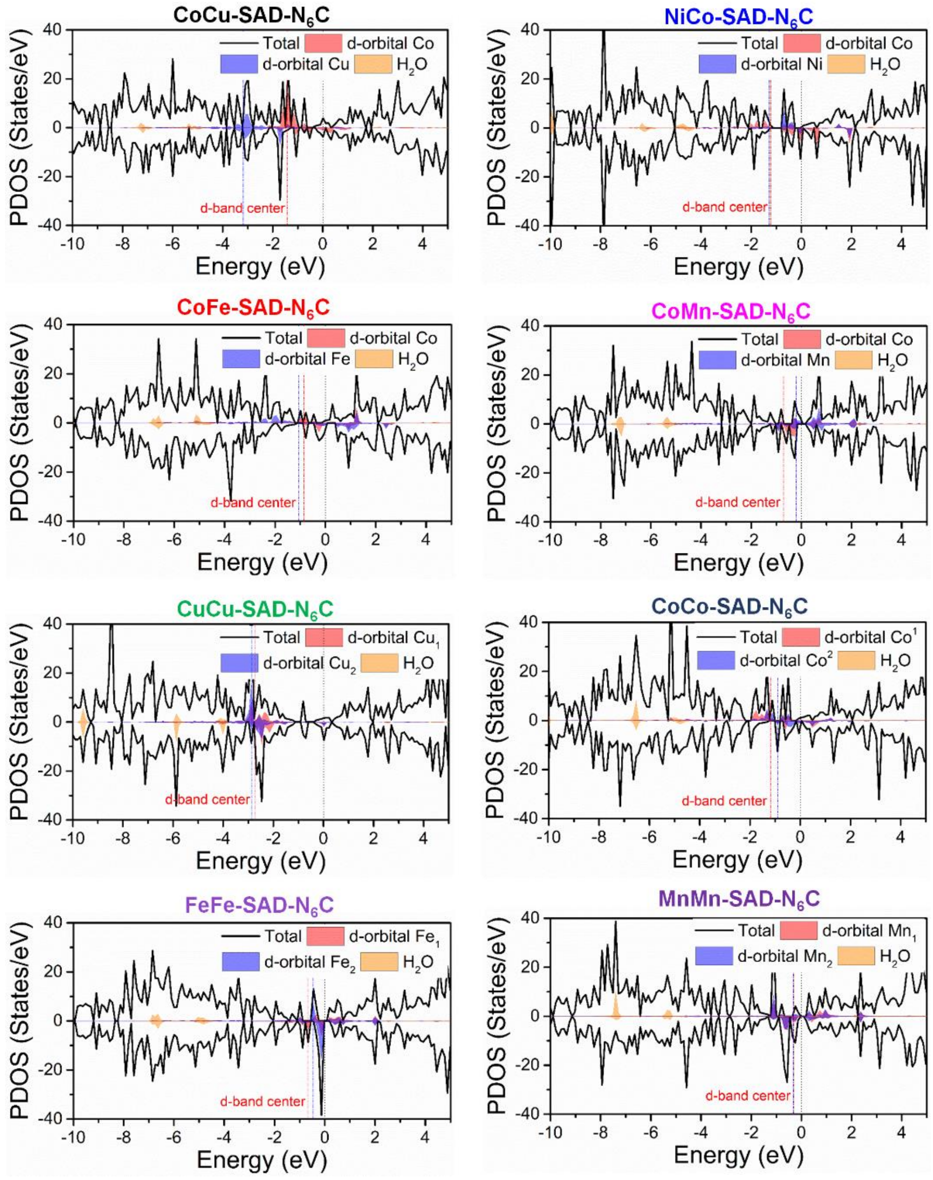

Supplementary Figure 3 | The spin polarized plots of adsorbed H_2O and d orbitals of metal atoms in TM-SAD-N_6C.

Supplementary Discussion 1.

	$d_{H2O\text{-}Ni}$ (Å)	Δq (*H_2O)	ICOHP	E_{ads} (*H_2O)
**H_2O@Ni NPs**	2.16	-0.19	-0.34	-1.00
**H_2O@NiNi-SAD-N_6C**	3.32	-0.07	-0.03	-0.28

Distance between adsorbed H_2O and Ni active site ($d_{H2O\text{-}Ni}$), calculated adsorption energy (E_{ads}) of H_2O, integrated crystal orbital Hamilton population (ICOHP) and Mulliken charge transfer (Δq) from an active atom into the adsorbed H_2O on NiNi-SAD-N_6C and Ni-NPs.

The integrated crystal orbital Hamilton population COHP (ICOHP) was calculated to examine interaction in Ni−H_2O bond quantitatively. It should be noted that more positive ICOHP value closer to the Fermi level indicated weaker covalent interaction between atoms. As shown in Supplementary Discussion 1, $E_{ads(*H2O)}$ increased as ICOHP shifted away from the Fermi level, suggesting that Ni-NPs exhibited stronger adsorption of H_2O molecule compared to NiNi-SAD. The weak Ni−H_2O bond in NiNi-SAD-N_6C could be attributed to fewer electrons in the valance state of Ni bonding with H_2O, and therefore the strength of adsorption with H_2O was weak.

Supplementary Table 2. **Comparison of different calculated energies among various TM-SAD-N_6C systems**. **a**, D-band center of the TM-SAD-N_6C systems with H* and H_2O* corresponding to the Gibbs adsorption free energy of the H* and energy barrier of water dissociation, respectively. **b**, The water adsorption energy and kinetic barrier of water splitting on six TM-SAD-N_6C (CoCr, CoMo, CoZn, CrCr, MoMo, and ZnZn) catalysts and free energy of *H and *OH.

a

	D-band center of SAD systems with H* (eV)	ΔG_{H*} (eV)	D-band center of SAD systems with H_2O* (eV)	E_{split} (eV)
**CoCu**	-2.16	0.126	-1.67	3.33
**NiCo**	-1.28	-0.34	-0.87	2.52
**CoFe**	-0.88	-0.121	-1.07	3.11
**CoMn**	-0.78	-0.46	-0.97	2.55
**CuCu**	-2.30	0.783	-2.82	4.22

**CoCo**	-0.86	-0.355	-1.50	2.94
**FeFe**	-0.90	-0.47	-1.05	2.64
**MnMn**	-0.16	-0.135	-0.89	2.76

b

	**E_{ads} (*H_2O) (eV)**	**Kinetic barrier (eV)**	**$\|\Delta G_{*OH}\|$**	**$\|\Delta G_{*H}\|$**
**CoCr**	-0.515	3.854	0.801	0.240
**CoMo**	-0.256	inactive	--	--
**CoZn**	-0.532	2.832	0.649	0.057
**CrCr**	-1.388	2.480	1.058	0.252
**MoMo**	-0.515	3.100	0.102	0.444
**ZnZn**	-0.464	2.827	0.104	0.249

As summarized in Supplementary Table 2b, all five dimers showed favorable H_2O adsorption with negative $E_{ads(*H2O)}$ values (exception for CoMo-SAD-N_6C) and rather easy H_2O dissociation with a relatively low kinetic barrier. Remarkably, CrCr-SAD-N_6C proved to be one of the most potential candidates with the lowest H_2O adsorption (-1.388 eV) and kinetic energy barrier for H_2O dissociation (2.480 eV). However, its theoretical overpotential was very high due to the higher free energy of OH* desorption (1.06 eV) compared to NiCo-SAD (0.46 eV). Therefore, NiCo-SAD-N_6C is still the most promising potential candidate for alkaline-HER.

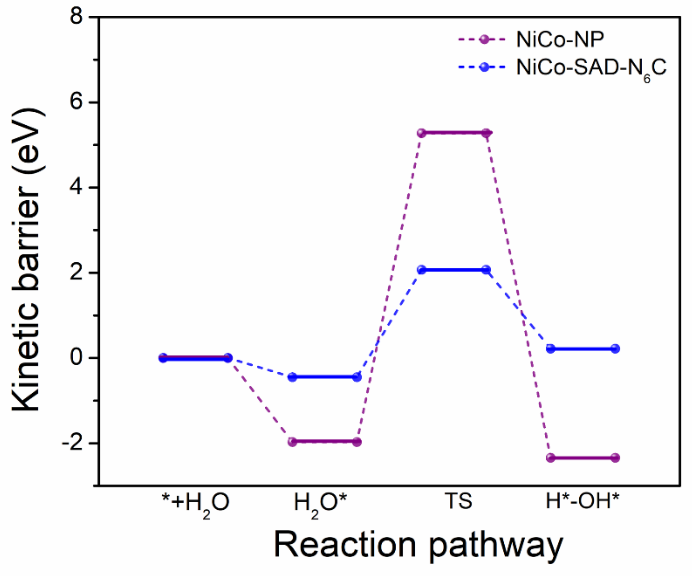

Supplementary Figure 4 | Comparison of minimum energy pathways of water splitting reactions on NiCo-SAD-N_6C and NiCo-NP.

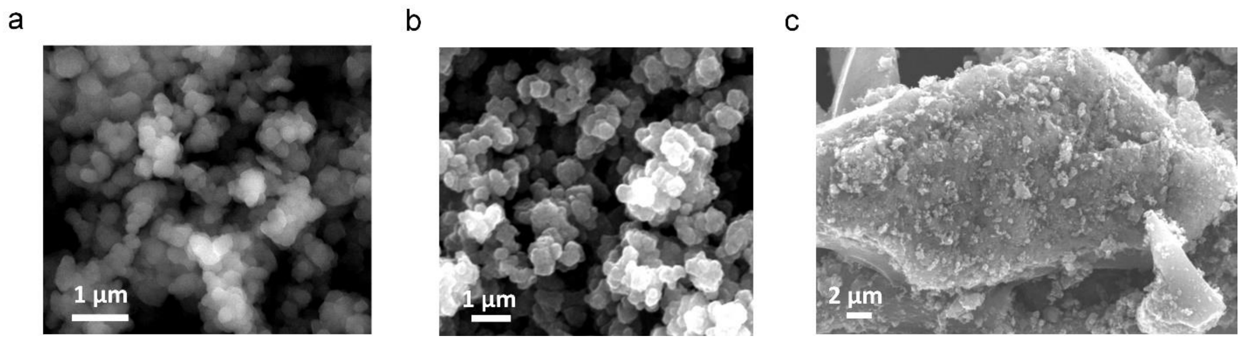

Supplementary Figure 5 | FESEM images of various samples. a, b, and **c** Field-emission scanning electron microscopy images of Ni^{2+}-Co^{2+}@Polydopamine, NiCo-NP-NC and NiCo-SAD-NC, respectively.

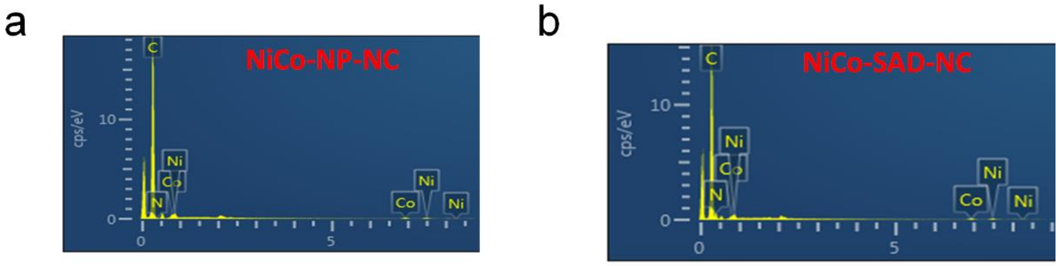

Supplementary Figure 6 | EDS patterns of various samples. a, and **b,** EDS pattern of the NiCo-NP-NC, and NiCo-SAD-NC, respectively.

Supplementary Table 3. **Atomic % of elements and Ni : Co ratio obtained from EDS and XPS analysis.**

Sample	EDS			Ni : Co (EDS)		XPS			Ni : Co (XPS)	
	Ni (at%)	Co (at%)	N (at%)	Expt.	Obser.	Ni (at%)	Co (at%)	N (at%)	Expt.	Obser.
NiCo-SAD-NC	0.79	0.88	17.41	1	0.9	0.55	0.529	18.1	1	1.03
NiCo-NP-NC	0.84	0.82	0.95	1	1.02	0.87	0.89	0.93	1	0.98
Ni-SA-NC	1.59	-	18.63	-	-	1.78	-	17.09	-	-
Co-SA-NC	-	1.64	16.68	-	-	-	1.57	16.96	-	-
NiCo-SAD-NC (1:2)	0.58	1.25	14.67	0.5	0.46					
NiCo-SAD-NC (2:1)	1.28	0.6	17.3	2	2.1					
NC	-	-	15.9	-	-	-	-	17.52	-	-

Supplementary Table 4. **Weight % of metal loading obtained from EDS and ICP-AES analysis**.

Sample	EDS				ICP-AES			Average (wt%) from EDS and ICP-AES
	Ni (wt%)	Co (wt%)	Pt (wt%)	Ni + Co (wt%)	Ni (wt%)	Co (wt%)	Ni + Co (wt%)	
NiCo-SAD-NC	3.94	3.56	-	7.5	3.719	3.331	7.05	7.28
NiCo-NP-NC	4.18	4.09	-	8.27	3.675	3.558	6.83	7.55
Pt-SA	-	-	4.5	-	-	-	-	-

Due to the introduction of additional N using dicyandiamide to trap the metal atoms, the total metal loading was slightly reduced while the N content increased for the NiCo-SAD-NC, slightly tuning the final composition.

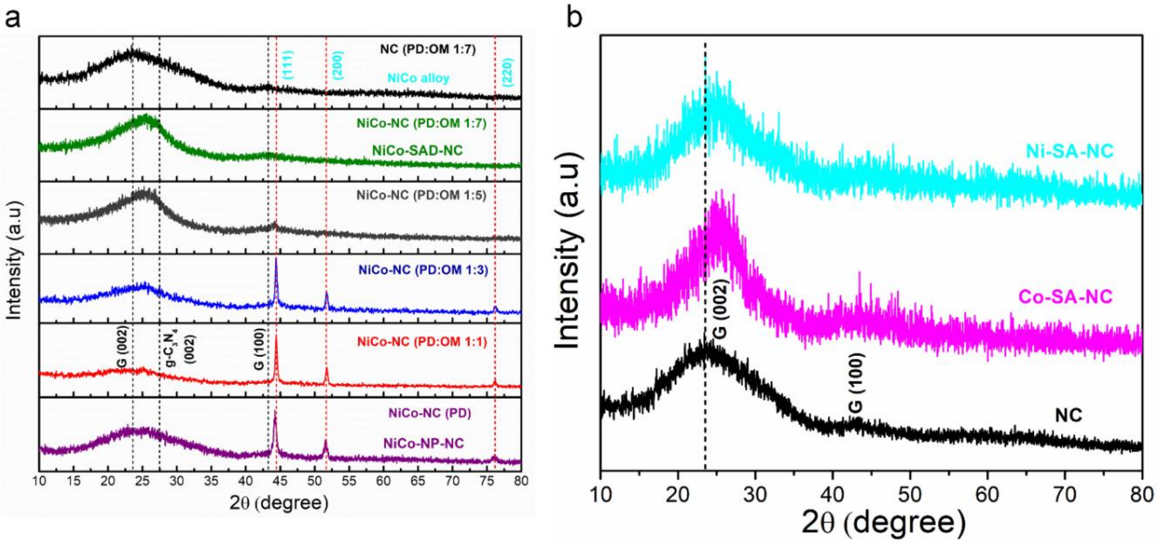

Supplementary Figure 7 | XRD analysis of as-prepared samples. a, XRD pattern of NiCo-NP-NC, NiCo-SAD-NC and other control sample obtained with different ratio of Ni^{2+}-Co^{2+}@Polydopamine (PD) precursor and dicyandiamide (organic molecule: OM). **b**, XRD pattern of NC, Co-SA-NC and Ni-SA-NC.

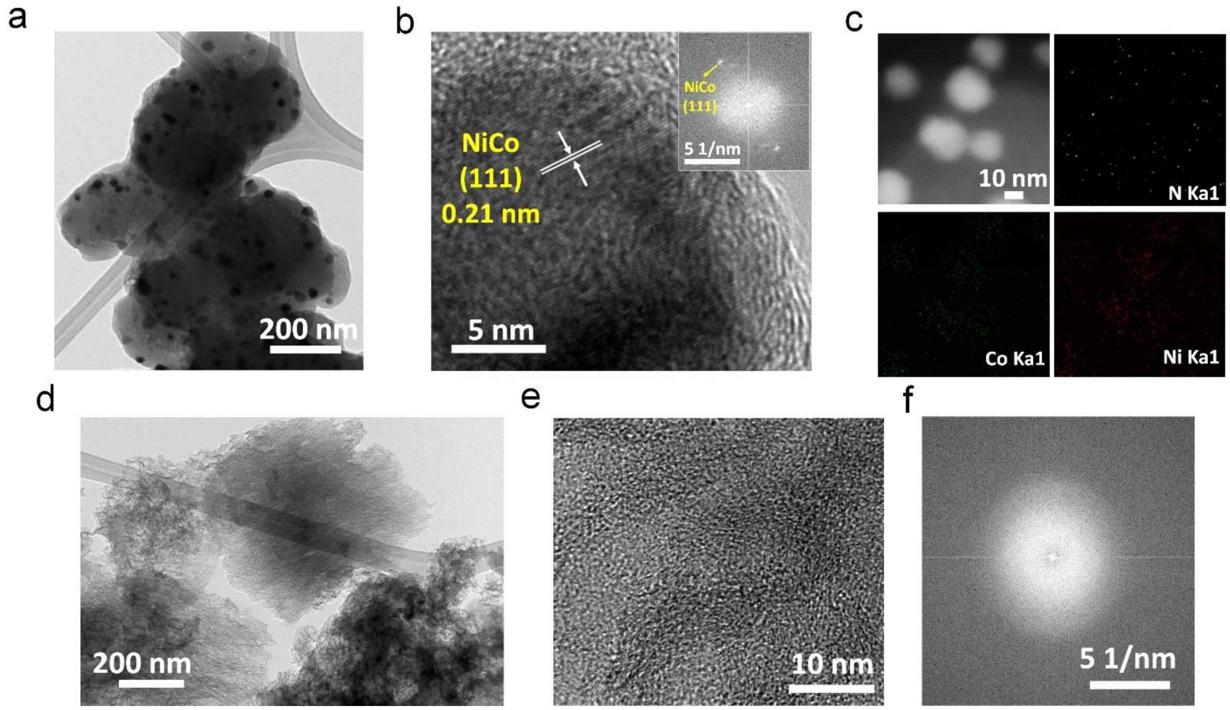

Supplementary Figure 8 | TEM, HRTEM, SAED pattern and elemental maps of as-prepared samples. a, b, and **c,** TEM, HRTEM and STEM-HAADF images with the corresponding element maps (N, Co, Ni), respectively, of NiCo-NP-NC. The inset in **b** shows the corresponding SAED pattern. **d, e,** and **f,** TEM, HRTEM image, and SAED pattern, respectively, of NiCo-SAD-NC.

The HRTEM image with the corresponding SAED pattern (Supplementary Fig. 8e, f) of NiCo-SAD-NC additionally confirmed the absence of any metal aggregations in the form of small clusters, consistent with the XRD pattern, suggesting that the metal atoms were atomically dispersed on the carbon support.

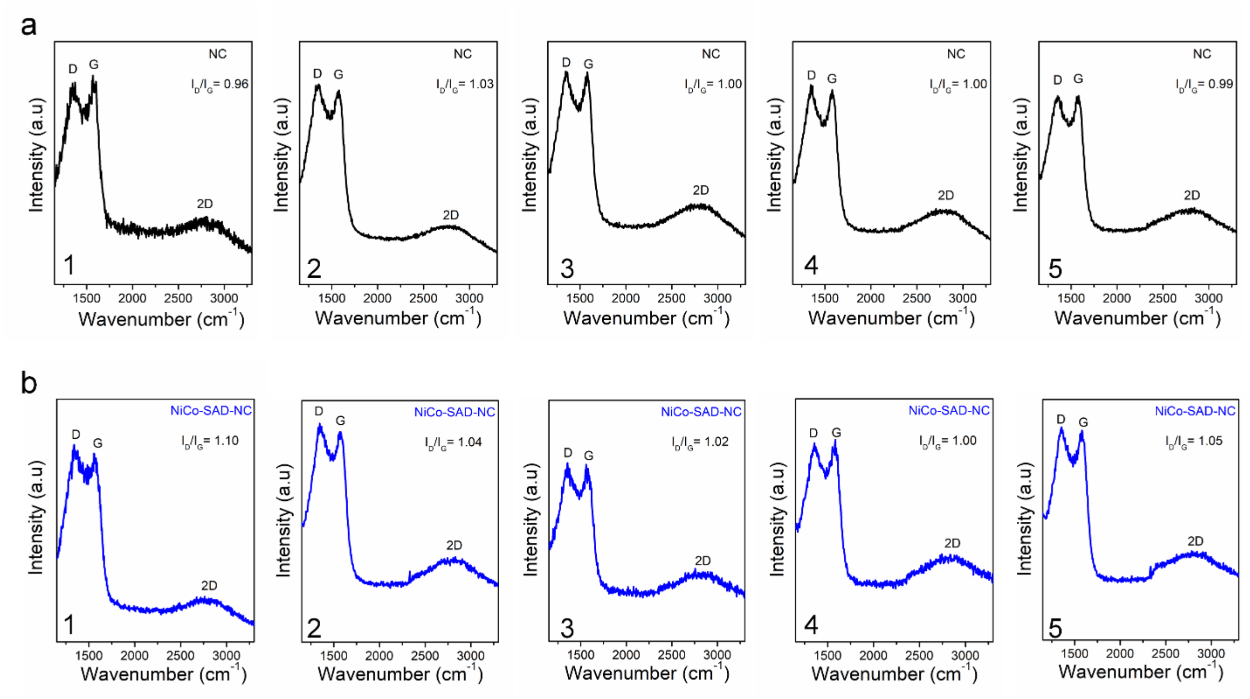

Supplementary Figure 9 | Raman analysis of NC and NiCo-SAD-NC. a, and **b,** Statistical analysis of Raman spectra of NC and NiCo-SAD-NC, respectively.

The Raman spectra of both NC and NiCo-SAD-NC showed a characteristic D band at 1338 cm^{-1} and G band at 1591 cm^{-1}, corresponding to carbon lattice defect and sp^2-hybridized carbon atoms, respectively, consistent with the XRD results (Supplementary Fig. 9).

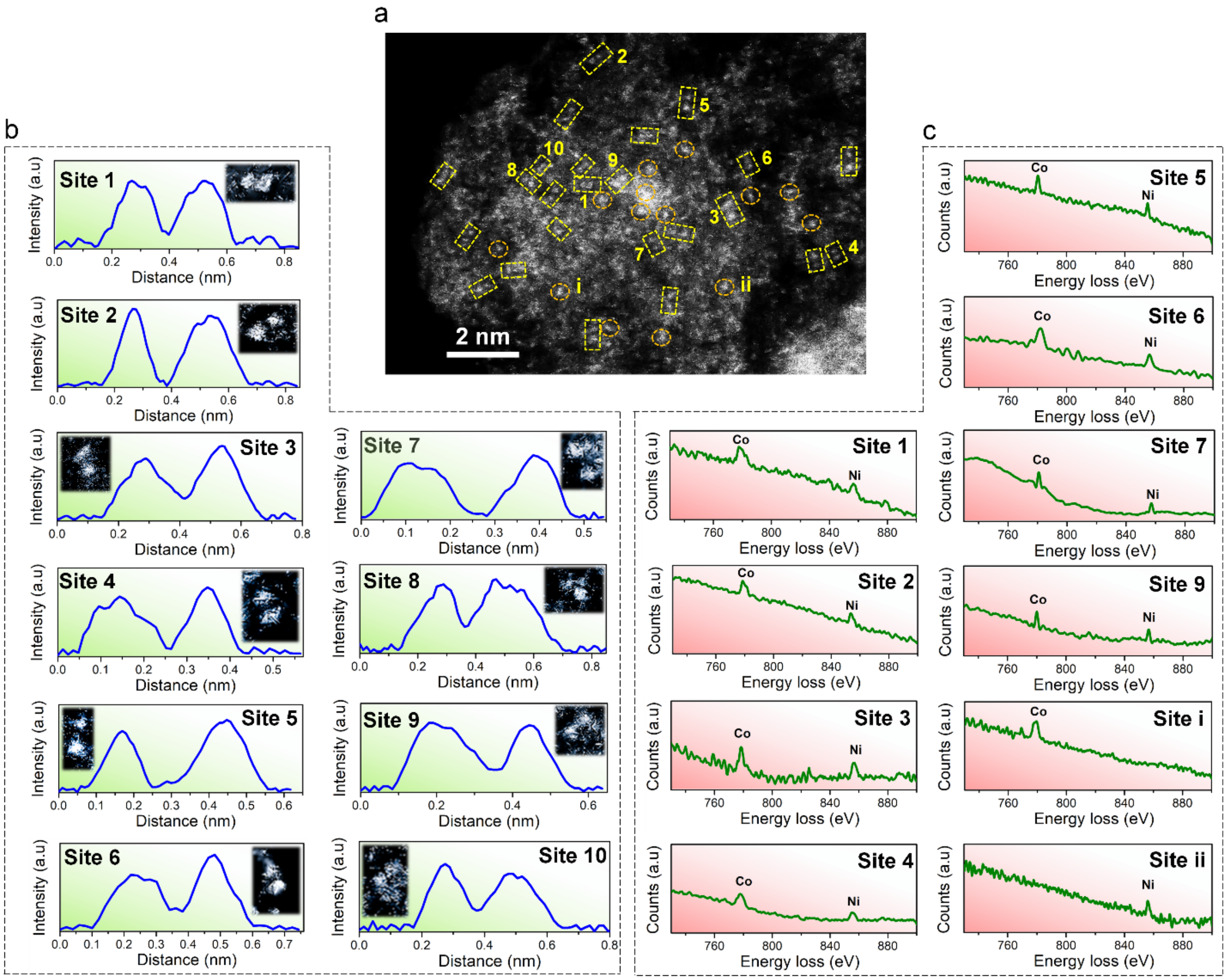

Supplementary Figure 10 | HAADF-STEM image along with EEL spectrums and intensity profiles of NiCo-SAD-NC. a, Aberration-corrected HAADF-STEM image of the NiCo-SAD-NC. The yellow squares in (**a**) shows the dimer sites and some of single Ni/Co-atom sites are highlighted by orange circles. **b**, The intensity profile obtained from site 1-10 showing the distance between Ni and Co in the observed dimers at the atomic scale. **c**, The corresponding EEL spectrum obtained from site 1-7, 9, and site i, ii showing the Ni and Co coordination in the dimer along with few Ni/Co single-atom sites.

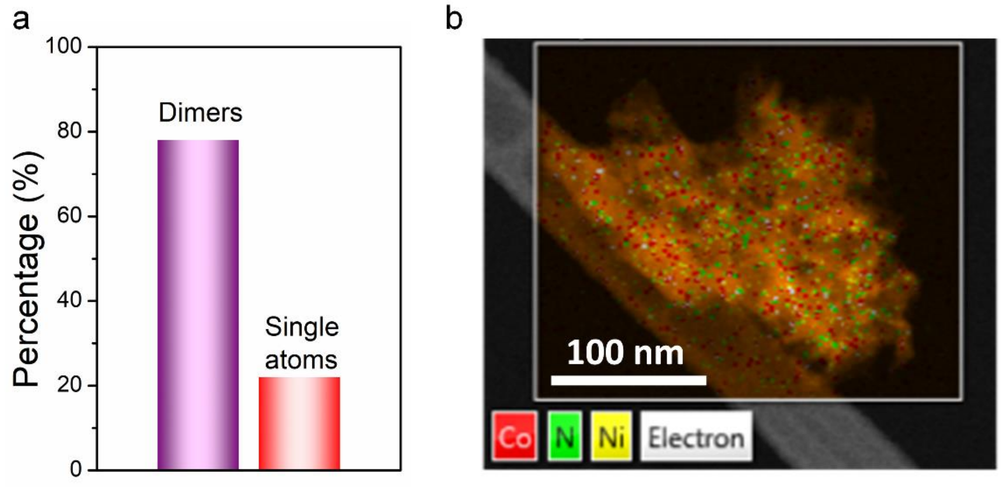

Supplementary Figure 11 | Ratio of dimer sites and EDS map of NiCo-SAD-NC. a, Distribution histogram showing the ratio between dimers, and single atoms. **b,** Overlapping EDS map of NiCo-SAD-NC showing the uniform distribution of Ni, Co and N atoms.

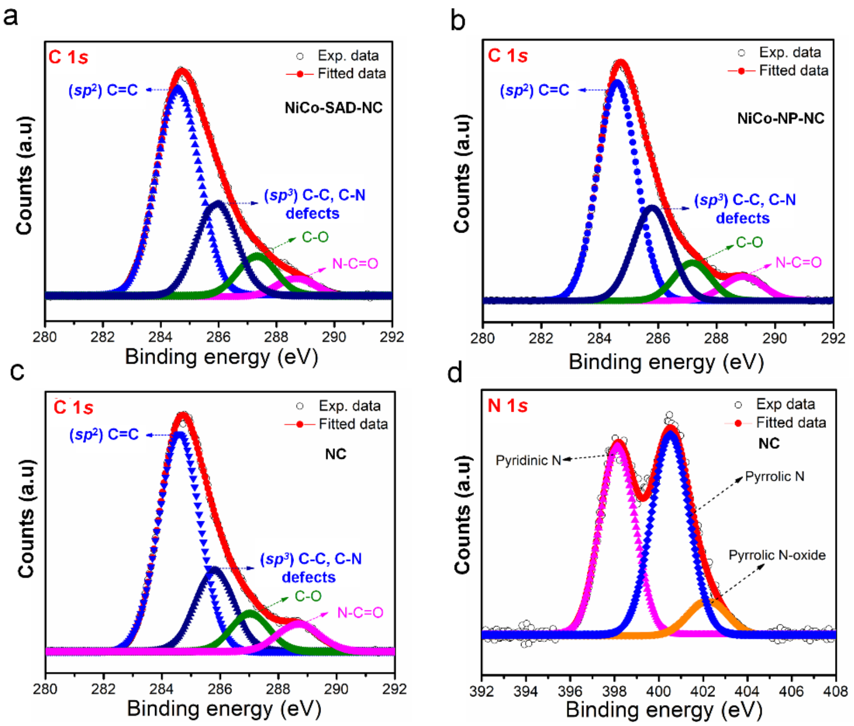

Supplementary Figure 12 | XPS spectra of as-prepared samples. a, b, and **c,** Fitted deconvoluted C *1s* XPS spectra of NiCo-SAD-NC, NiCo-NP-NC, and NC, respectively. **d,** Fitted deconvoluted N *1s* XPS spectra of NC.

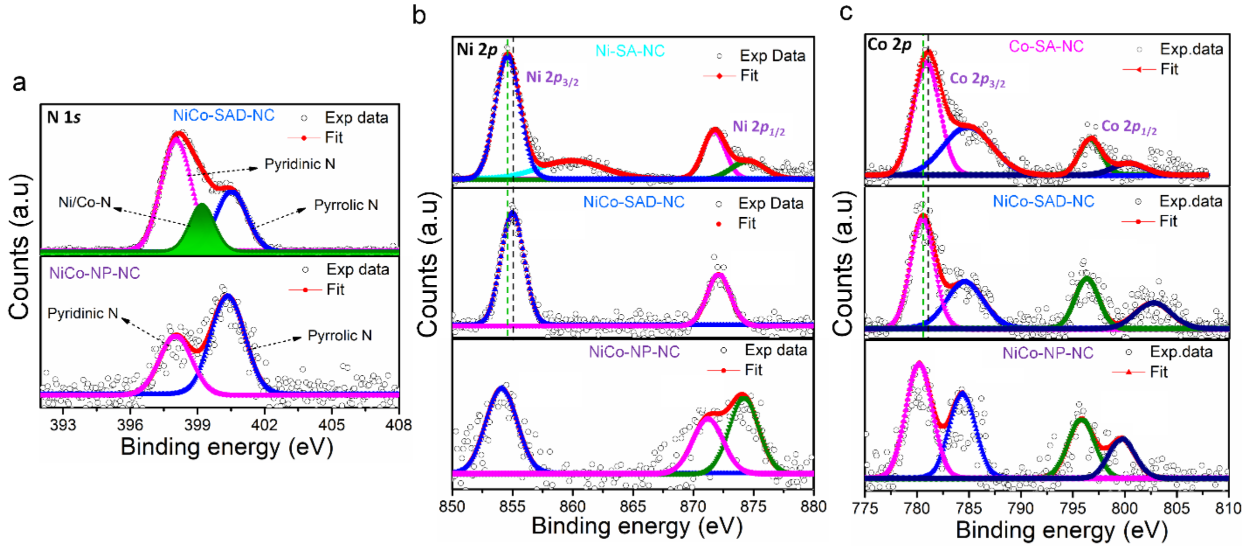

Supplementary Figure 13 | XPS spectra of as-prepared samples. a, Fitted deconvoluted N *1s* XPS spectra of NiCo-SAD-NC and NiCo-NP-NC. **b**, and **c**, Fitted deconvoluted Ni *2p* and Co *2p*, respectively, XPS spectra of NiCo-SAD-NC, NiCo-NP-NC, Co-SA-NC, and Ni-SA-NC.

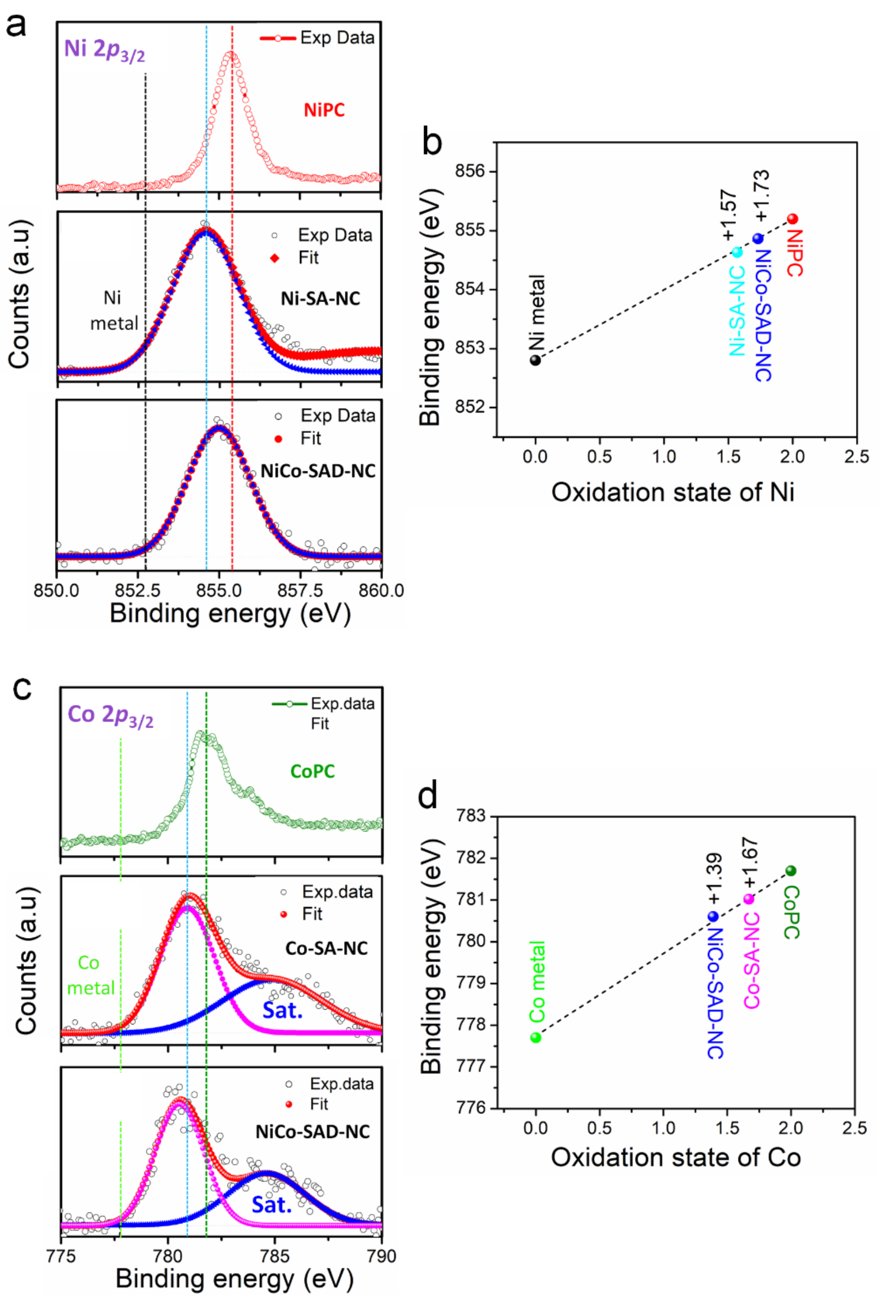

Supplementary Figure 14 | XPS spectra and oxidation states of as-prepared samples. a, and **b**, Fitted deconvoluted Ni $2p_{3/2}$ XPS spectra and the Ni oxidation states analyzed by the XPS peak position, respectively, of NiCo-SAD-NC, and Ni-SA-NC with reference samples. **c**, and **d**, Fitted deconvoluted Co $2p_{3/2}$ XPS spectra and the Co oxidation states analyzed by the XPS peak position, respectively, of NiCo-SAD-NC, and Co-SA-NC with reference samples.

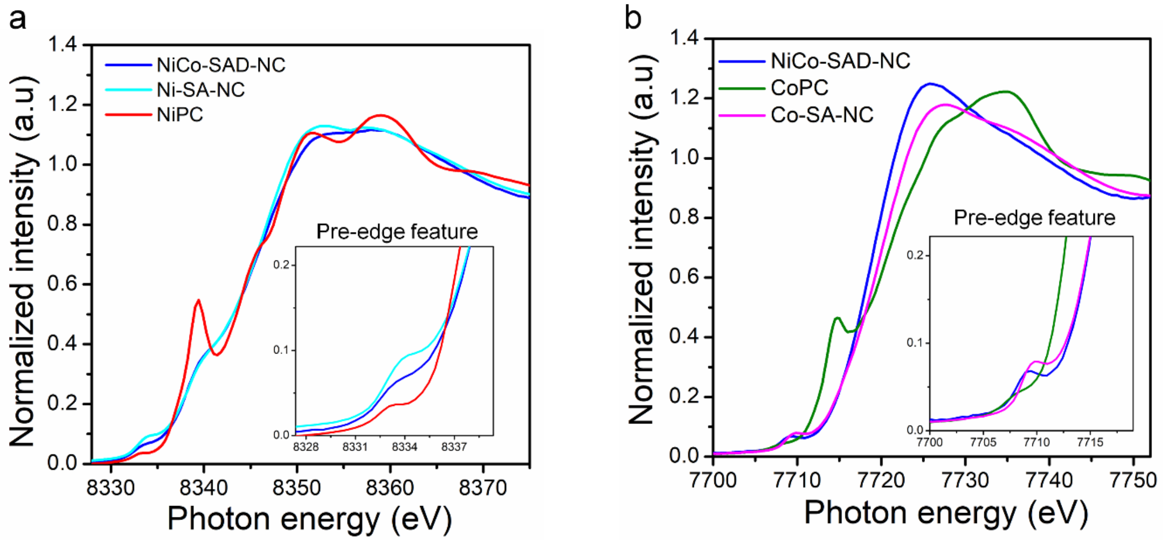

Supplementary Figure 15 | XANES spectra and pre-edge features of as-prepared samples. a, Experimental Ni K-edge XANES spectra, of NiCo-SAD-NC with Ni-SA-NC and NiPC (Inset in **a** show the pre-edge feature). **b**, Experimental Co K-edge XANES spectra, of NiCo-SAD-NC with Co-SA-NC and CoPC (Inset in **b** show the pre-edge feature).

The increased intensity of the first pre-edge peak at 8333.8 and 7709 eV in the Ni and Co K-edge XANES spectra, respectively, of NiCo-SAD-NC compared to NiPC and CoPC were ascribed to the increased dipole allowed transition ($1s \rightarrow 4p$), which occurred due to the mixing of 3*d* and 4*p* orbitals as a result of the distorted D_{4h} symmetry. In contrast, the intensity of the second pre-edge peak at 8339 and 7715 eV ($1s \rightarrow 4p_z$) in the Ni and Co K-edge XANES spectra, respectively, of NiCo-SAD-NC were reduced compared to the NiPC/CoPC, thus further confirming the distorted D_{4h} symmetry of the Ni and Co atoms centers in the NiCo-SAD-NC (Supplementary Fig. 15).

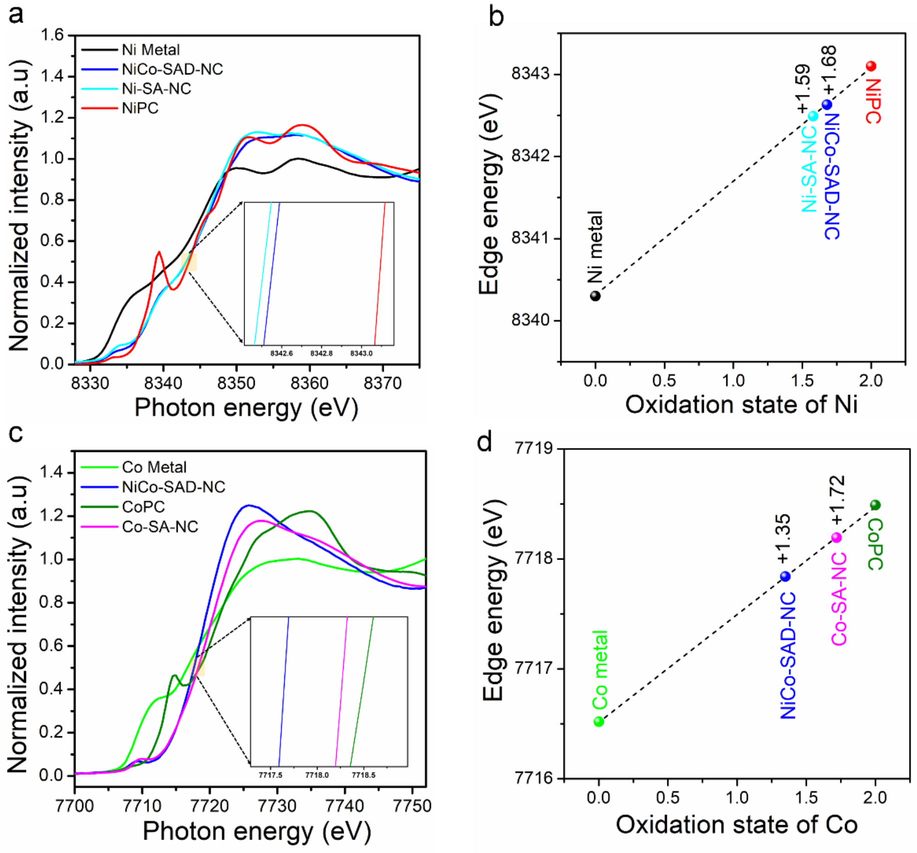

Supplementary Figure 16 | XANES spectra and oxidation states of as-prepared samples. a, and **b**, Experimental Ni K-edge XANES spectra of NiCo-SAD-NC with reference samples and the Ni oxidation state analysis by corresponding XANES energy at half-edge jump, respectively. **c**, and **d**, Experimental Co K-edge XANES spectra of NiCo-SAD-NC with reference samples and the Co oxidation state analysis by corresponding XANES energy at half-edge jump, respectively.

Compared to the difference in the electronegativity between Ni and N in Ni-SA-NC, due to the larger difference in the electronegativity between Co and N in the Co-SA-NC, the Co oxidation state in Co-SA-NC was higher than the Ni in Ni-SA-NC, revealed from their respective XPS and XANES spectra. In the case of NiCo-SAD-NC, when Co at a higher oxidation state underwent strong electronic interaction with the Ni at a relatively lower oxidation state at the interface, then the electron transfer occurred from the Ni to Co site and the final oxidation state of Ni in NiCo-SAD-NC became higher than the Ni in Ni-SA-NC. Contrarily, the oxidation state of Co in NiCo-SAD-NC became lower than the Co in Co-SA-NC. Therefore, the electron transfer between the Ni and Co suggested the successful generation of the atomic interface in NiCo-SAD-NC.

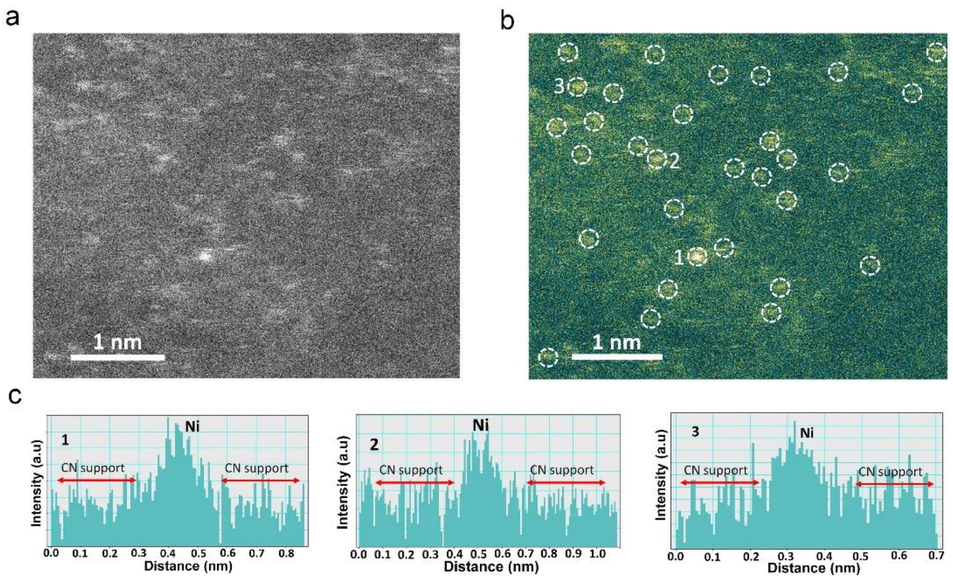

Supplementary Figure 17 | HAADF-STEM image and intensity profiles of Ni-SA-NC. a, and **b**, Aberration-corrected HAADF-STEM image of the Ni-SA-NC. The white circles in (**b**) shows the isolated Ni-SA uniformly dispersed. **c**, The intensity profile obtained at Ni-SA site 1, 2, and 3.

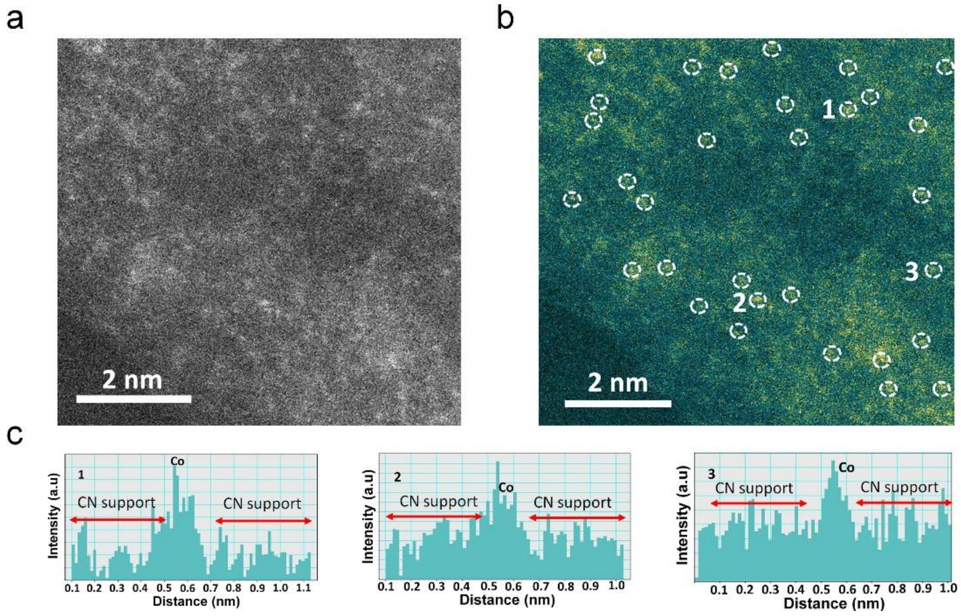

Supplementary Figure 18 | HAADF-STEM image and intensity profiles of Co-SA-NC. a, and **b**, Aberration-corrected HAADF-STEM image of the Co-SA-NC. The white circles in (**b**) shows the isolated Co-SA uniformly dispersed. **c**, The intensity profile obtained at Co-SA site 1, 2, and 3.

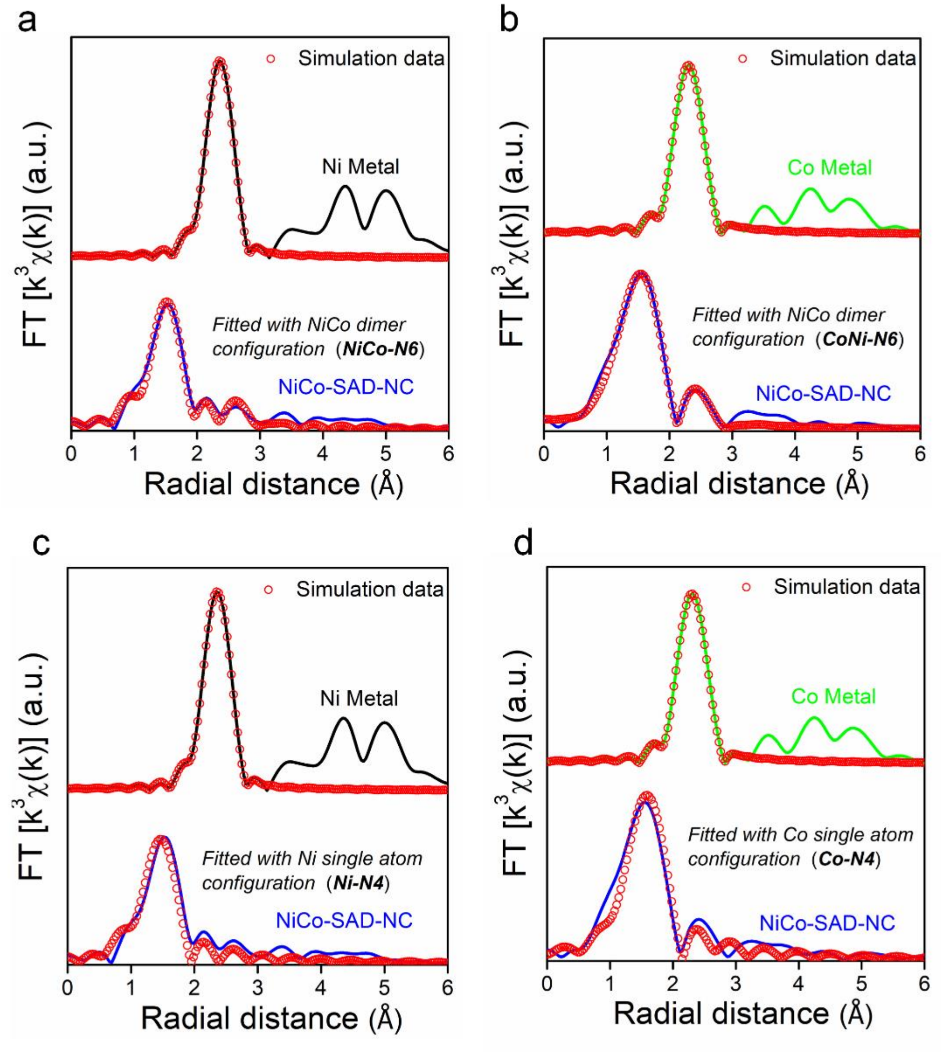

Supplementary Figure 19 | EXAFS fitting curve for NiCo-SAD-NC with dimer and single atom configuration. a, and **b,** The Ni K-edge, and Co K-edge FT-EXAFS, respectively, of NiCo-SAD-NC along with Ni/Co metal fitted with NiCo-N6 dimer configuration. **c,** and **d,** The Ni K-edge, and Co K-edge FT-EXAFS, respectively, of NiCo-SAD-NC along with Ni/Co metal fitted with Ni/Co-N4 single atom configuration.

Supplementary Table 5. **EXAFS fitting parameters at the Ni and Co K-edge for NiCo-SAD-NC and Ni/Co metal (S_0^2= 0.780 (Ni), 0.816(Co)).**

Sample	Shell	N^a	R(Å)^b	σ^2(Å^2)^c	ΔE_0(eV)^d	R factor
	**Co K-edge** (*fitted with CoNi-N6 dimer configuration*)					
Co Metal	Co-Co	12	2.49	0.0060	6.6	0.0002
NiCo-SAD-NC	Co-N	3.1	2.01	0.0018	-4.5	0.0001
	Co-Ni	0.6	2.55	0.0126		
	**Ni K-edge** (*fitted with NiCo-N6 dimer configuration*)					
Ni Metal	Ni-Ni	12	2.48	0.0059	6.3	0.0001
NiCo-SAD-NC	Ni-N	3.3	1.86	0.0039	-4.8	0.0016
	Ni-Co	0.7	2.55	0.0121		

	**Co K-edge** (*fitted with Co-N4 single atom configuration*)					
NiCo-SAD-NC	Co-N	4.0	2.03	0.0094	-2.1	0.0082
	**Ni K-edge** (*fitted with Ni-N4 single atom configuration*)					
NiCo-SAD-NC	Ni-N	4.0	1.83	0.0064	-8.3	0.0199

$^a N$: coordination numbers; $^b R$: bond distance; $^c\sigma^2$: Debye-Waller factors; ^{d} ΔE_0: the inner potential correction. R factor: goodness of fit. S_0^2 was set to 0.816 for Co and 0.780 for Ni, according to the experimental EXAFS fit of Co and Ni metal reference by fixing CN as the known crystallographic value.

The NiCo-SAD-NC FT-EXAFS spectra were fitted with NiCo-N6 dimer configuration with R-factor values of 0.0001 and 0.0016 for the Co and Ni K-edge EXAFS fitting, respectively, which were much smaller than the R-factor values of 0.0082 and 0.0199 for the Co and Ni K-edge EXAFS fitting, respectively, when fitted with Co/Ni-N4 single atom configuration, suggesting that the NiCo-SAD-NC FT-EXAFS spectra were best fitted with the NiCo-N6 dimer configuration, confirming that a significant amount of dimer structure was present in the prepared NiCo-SAD-NC material, consistent with the HAADF-STEM results.

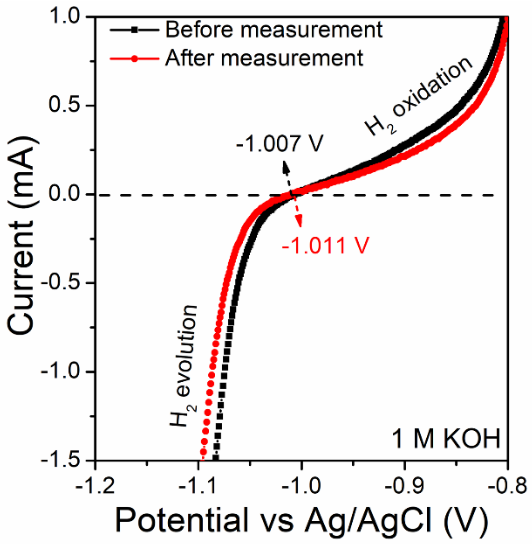

Supplementary Figure 20 | Ag/AgCl reference electrode calibration in H_2 saturated 1 M KOH solution.

The calibration of Ag/AgCl reference electrode was done in H_2 saturated 1 M KOH solution with Pt mesh as both working and counter electrode. Th LSV curve were obtained at a scan rate of 3 mV s^{-1}. The potential at zero current is the reversible potential for H_2 evolution/oxidation. Initially, the zero-point current is at -1.007 V correspond to $E_{RHE} \sim 0$ ($E_{RHE} = E_{Ag/AgCl} + 1$). Whereas, after measurement, the zero current remains at -1.011 V, suggesting that the reference electrode potential remains unaltered under the alkaline media.

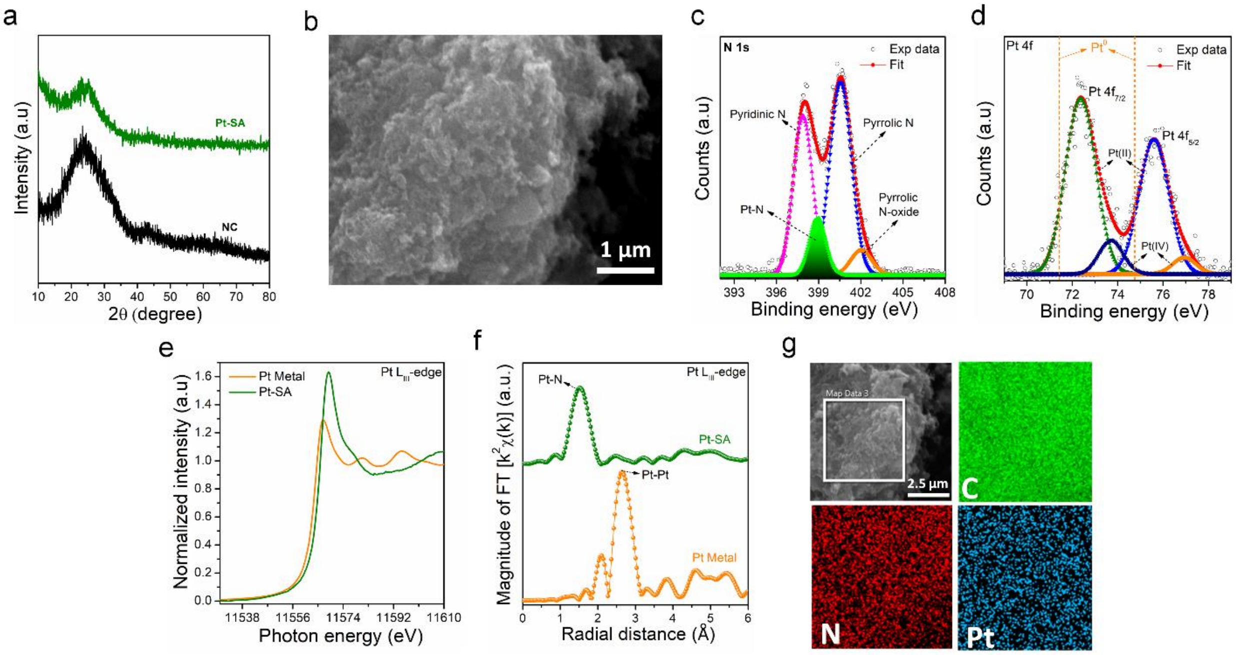

Supplementary Figure 21 | Structural analysis of Pt-SA. a, XRD pattern of NC and Pt-SA**. b**, **c**, and **d**, FESEM, fitted deconvoluted N *1s* and Pt *4f* XPS spectra, respectively, of Pt-SA. **e**, and **f**, Experimental Pt L_{III}-edge XANES and FT-EXAFS spectra of Pt-SA and Pt metal. **g**, EDS mapping of Pt-SA showing the uniform dispersion of C (green), N (red) and Pt (blue).

The matching XRD pattern of Pt-SA with NC confirms the absence of Pt-based clusters/nanoparticles, whereas the FESEM image revealed the fluffy porous morphology of Pt-SA (Supplementary Fig. 21a,b). While the N 1s XPS spectra confirmed the bonding between N and Pt atoms, Pt 4f XPS spectra revealed that the Pt in Pt-SA exhibited a higher oxidation state compared to Pt^0 due to higher electronegativity of coordinating N (Supplementary Fig. 21c,d). The positive shift of the Pt L_{III}-edge XANES spectra of Pt-SA compared to Pt metal also confirmed the higher oxidation state of Pt (Supplementary Fig. 21e). The Pt L_{III}-edge FT-EXAFS spectra of Pt-SA showed single predominant peaks in the R space at around 1.52 Å assigned to Pt-N bonds confirming that all Pt atoms in Pt-SA were atomically dispersed and stabilized by N coordination (Supplementary Fig. 21f). The EDS mapping confirmed the uniform distribution of Pt, C, and N in the Pt-SA catalyst (Supplementary Fig. 21g).

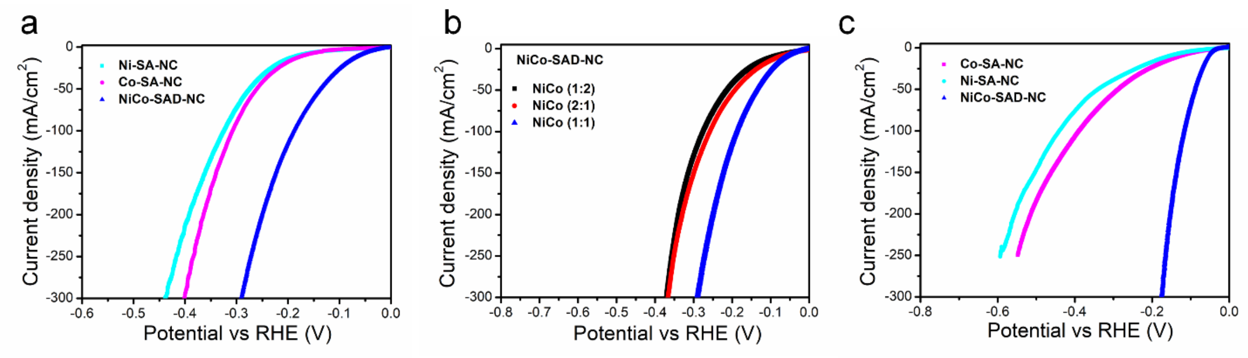

Supplementary Figure 22 | HER LSV polarization curves of various as-prepared catalysts. **a,** and **b**, HER LSV polarization curve of NiCo-SAD-NC with Ni/Co-SA-NC and different ratio of Ni to Co, respectively, in 1 M KOH. **c**, HER LSV polarization curve of NiCo-SAD-NC with Ni/Co-SA-NC in 0.5 M H_2SO_4.

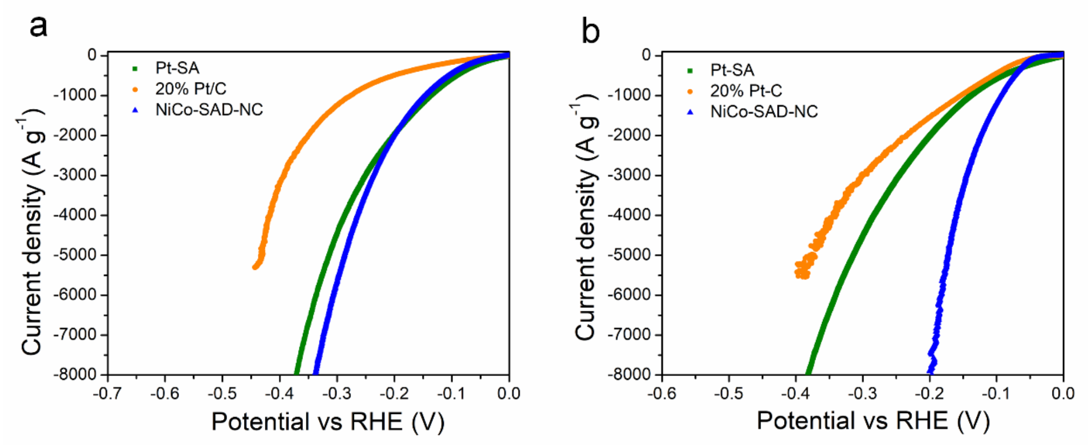

Supplementary Figure 23 | Mass activity comparison of NiCo-SAD-NC with Pt-SA and Pt/C. **a,** and **b**, Mass activity of NiCo-SAD-NC with 20% Pt-C and Pt-SA in 1 M KOH and 0.5 M H_2SO_4, respectively.

Supplementary Discussion 2:

The most stable water absorption geometries are illustrated in below table.

	**Δμ**	**Δδ (e^-)**
**CuCo**	0.02	-0.036
**FeCo**	-0.05	0.922
**MnCo**	-0.33	0.446
**NiCo**	0.03	-0.158

The difference in electronegativity ($\Delta\mu = M_\mu - Co_\mu$) and Mulliken charge difference ($\Delta\delta = M_\delta - Co_\delta$) on MCo-SAD-NC.

Notably, H_2O tends to be adsorbed on the top site of the metal atom with lower electronegativity corresponding to a higher positive charge. Hence, it is adsorbed on the Fe/Mn top sites in the case of MnCo/FeCo-SAD-NC, and on the Co top site in the NiCo/CuCo-SAD-NC case. According to our results, NiCo-SAD-NC had the smallest water dissociation barrier of 2.52 eV. This means that the Co site in the NiCo-SAD-NC adsorbed the H_2O and facilitated the H-OH cleaving of the water molecules. After water dissociation, H* was preferably adsorbed on the higher electronegative metal sites (Ni) (Volmer step), and *OH remained on the lower electronegativity Co site, followed by proton dimerization to molecular H_2 from the Ni site (Heyrovsky step).

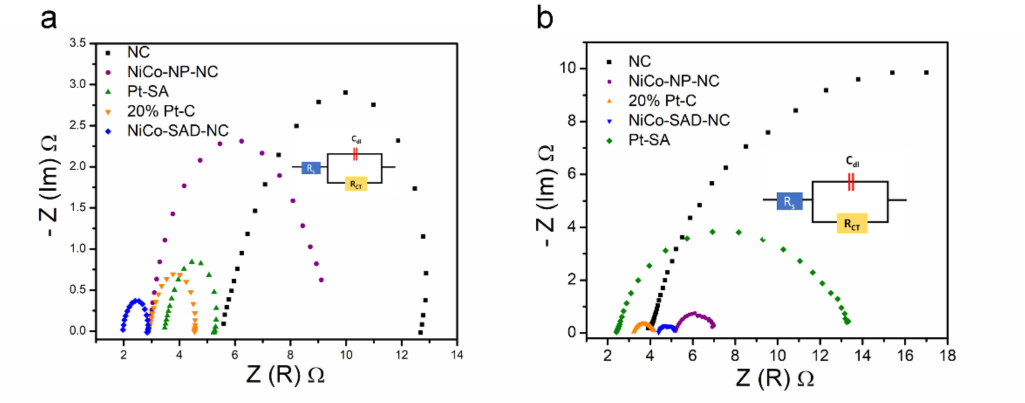

Supplementary Figure 24 | EIS analysis. a, and **b**, Nyquist plot at 464 mV overpotential in 1 M KOH and 290 mV overpotential in 0.5 M H_2SO_4, respectively (iR-uncorrected).

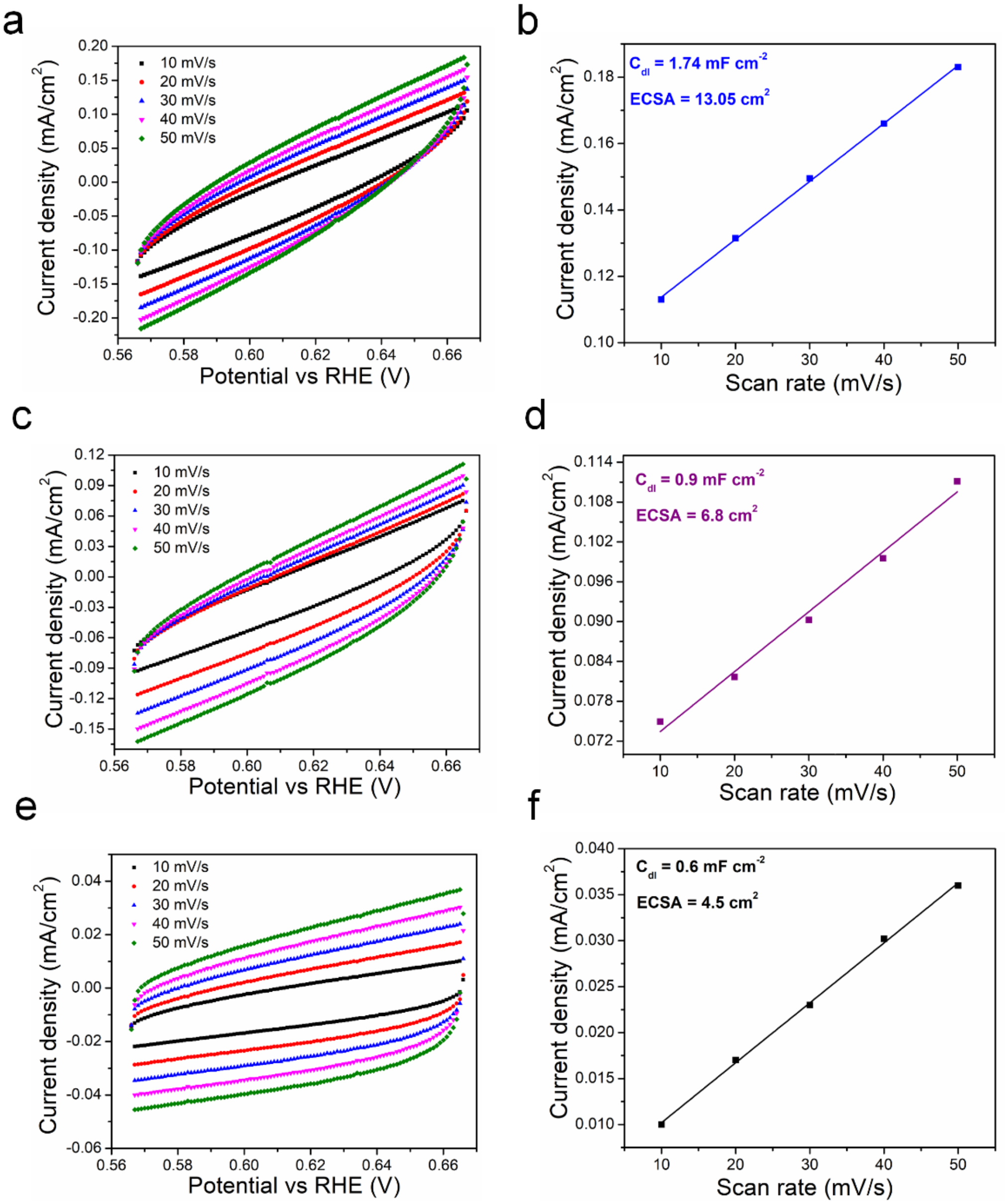

Supplementary Figure 25 | ECSA determination of as-prepared catalysts. a, c, and **e**, CV plots of NiCo-SAD-NC, NiCo-NP-NC, and NC, respectively, at different scan rates. **b**, **d**, and **f**, Current density (recorded at a fixed potential) as a function of scan rate for NiCo-SAD-NC, NiCo-NP-NC, and NC, respectively.

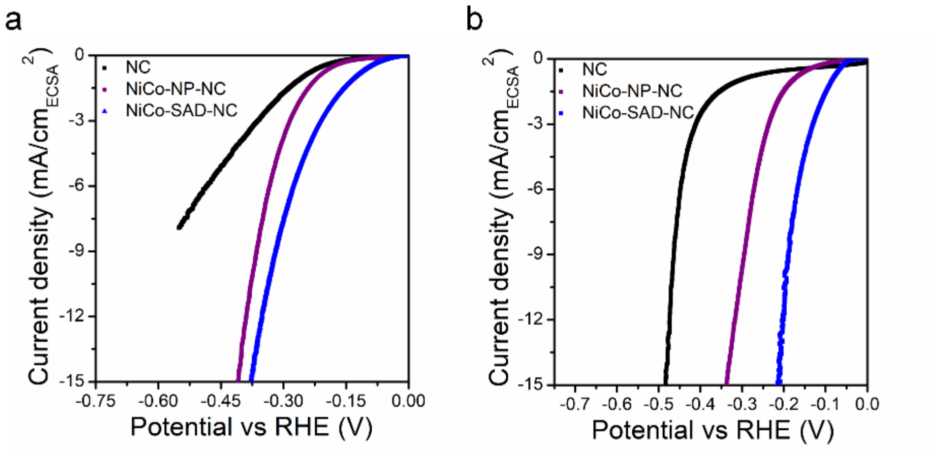

Supplementary Figure 26 | ECSA normalized LSV curves. a, and **b**, ECSA normalized HER LSV polarization curve in 1 M KOH and 0.5 M H_2SO_4, respectively.

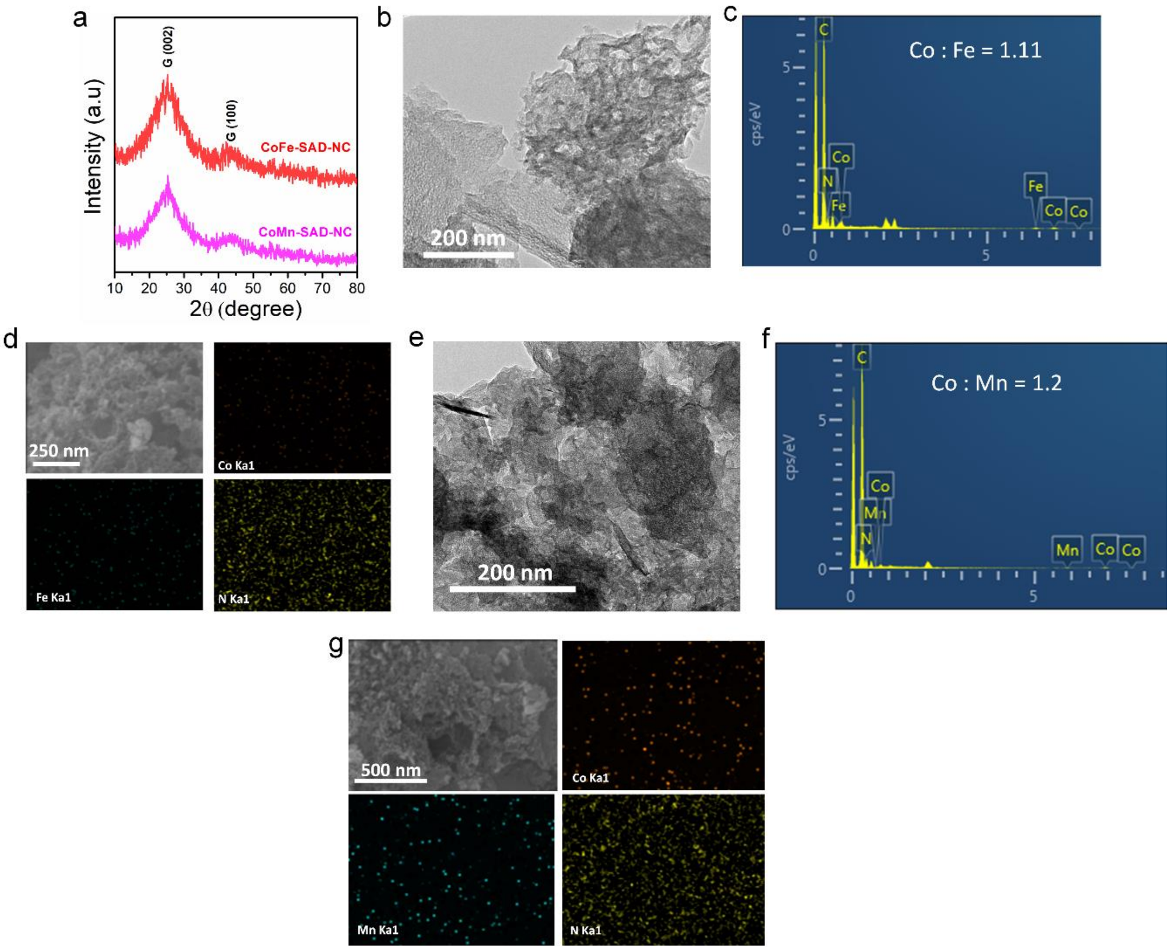

Supplementary Figure 27 | Structural characterizations of CoMn-SAD-NC and CoFe-SAD-NC. a, XRD pattern of CoMn-SAD-NC and CoFe-SAD-NC. **b**, **c,** and **d** TEM, EDS pattern and EDS mapping, respectively, of CoFe-SAD-NC. **e**, **f,** and **g** TEM, EDS pattern and EDS mapping, respectively, of CoMn-SAD-NC.

The XRD pattern of CoMn-SAD-NC and CoFe-SAD-NC in Supplementary Fig. 27a is similar to that of NC, suggesting the absence of any NPs, corroborated by the TEM images in Supplementary Fig. 27b,e. The EDS pattern along with EDS mapping confirmed the uniform distribution of atomically dispersed Co, Fe, and N in CoFe-SAD-NC and Co, Mn, and N in CoMn-SAD-NC (Supplementary Fig. 27c,d,f,g).

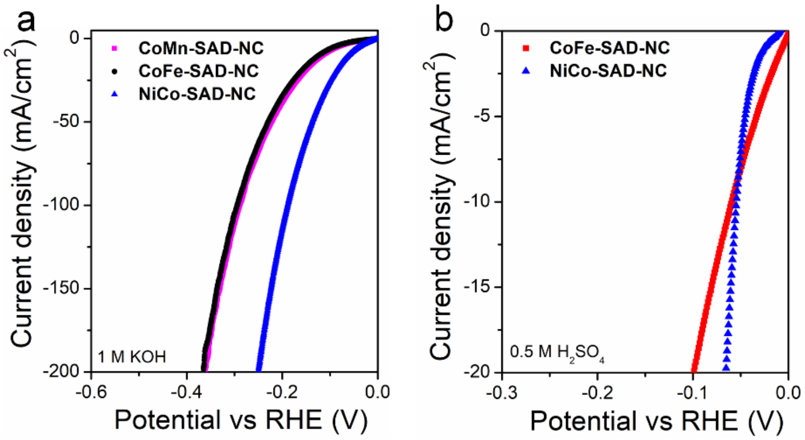

Supplementary Figure 28 | HER LSV polarization curves. a, HER LSV polarization curve of NiCo-SAD-NC with CoMn-SAD-NC and CoFe-SAD-NC, in 1 M KOH. **b**, HER LSV polarization curve of NiCo-SAD-NC with CoFe-SAD-NC in 0.5 M H_2SO_4.

Consistent with the DFT prediction, we experimentally found that the HER LSV polarization curve for the CoFe-SAD-NC and CoMn-SAD-NC in alkaline media were close to each other with CoMn-SAD-NC requiring an overpotential of 116.5 mV at -10 mA/cm^2 slightly better than CoFe-SAD-NC (124.9 mV) (Supplementary Fig. 28).

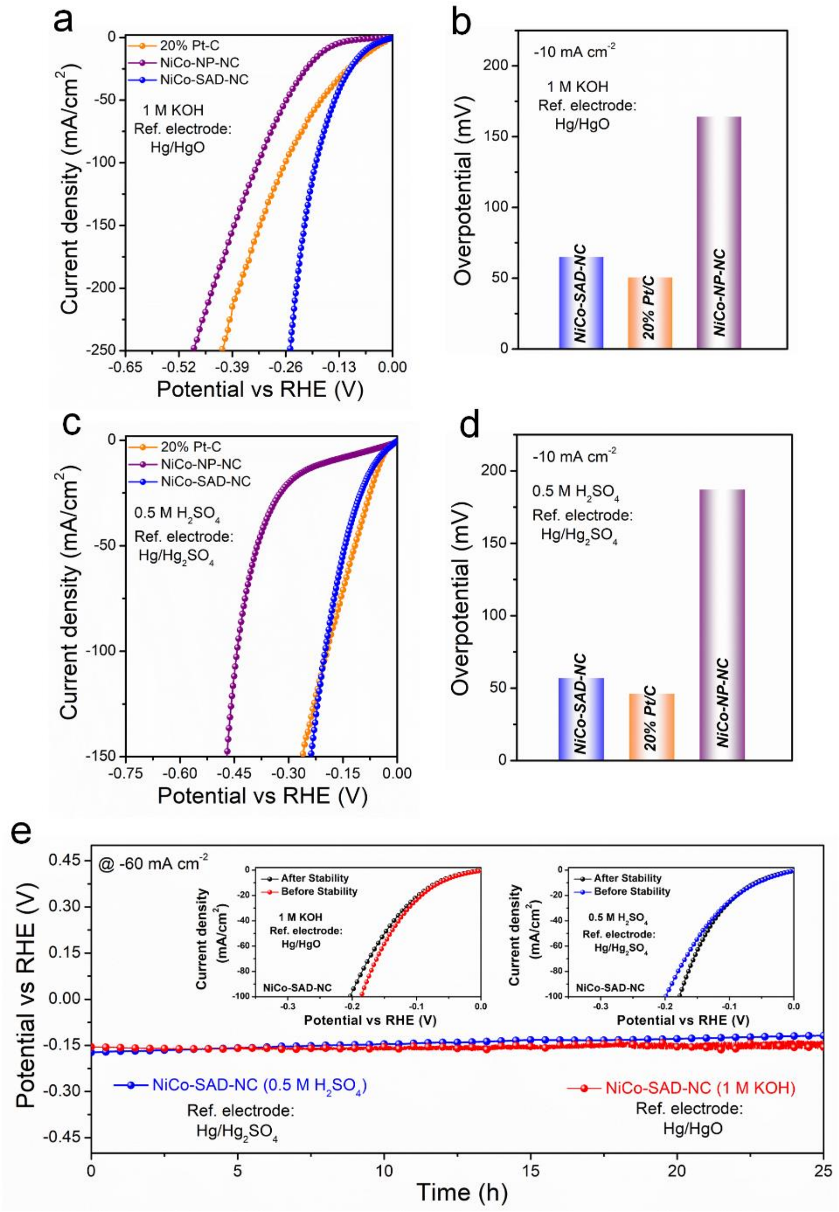

Supplementary Figure 29 | HER performance using different reference electrode. a, and **b**, HER LSV polarization curve and the overpotentials required to reach -10 mA cm^{-2} in 1 M KOH using Hg/HgO as reference electrode. **c,** and **d**, HER LSV polarization and the overpotentials required to reach -10 mA cm^{-2} in 0.5 M H_2SO_4 using Hg/Hg_2SO_4 as reference electrode. **e**, long-term chronopotentiometric stability test for NiCo-SAD-NC in 0.5 M H_2SO_4 (ref. electrode: Hg/Hg_2SO_4) and 1 M KOH (ref. electrode: Hg/HgO) at a current density of -60 mA cm^{-2}. The inset shows the LSV curves before and after the durability test.

As revealed in Supplementary Fig. 29a-d, the HER activity of NiCo-SAD-NC was still superior to NiCo-NP-NC and comparable to that of 20% Pt/C in alkaline and acidic media using Hg/HgO and

Hg/Hg_2SO_4 as reference electrodes, respectively, certifying the superior intrinsic activity of NiCo-SAD-NC. In addition, the NiCo-SAD-NC also demonstrated superior long-term stability for 25 h at -60 mA/cm^2 under both alkaline and acidic media using Hg/HgO and Hg/Hg_2SO_4 as reference electrodes, respectively, without any noticeable degradation (Supplementary Fig. 29e). The overlapping LSV polarization curves before and after the durability test confirmed the retention of the active sites and superior HER activity (Supplementary Fig. 29e, insets).

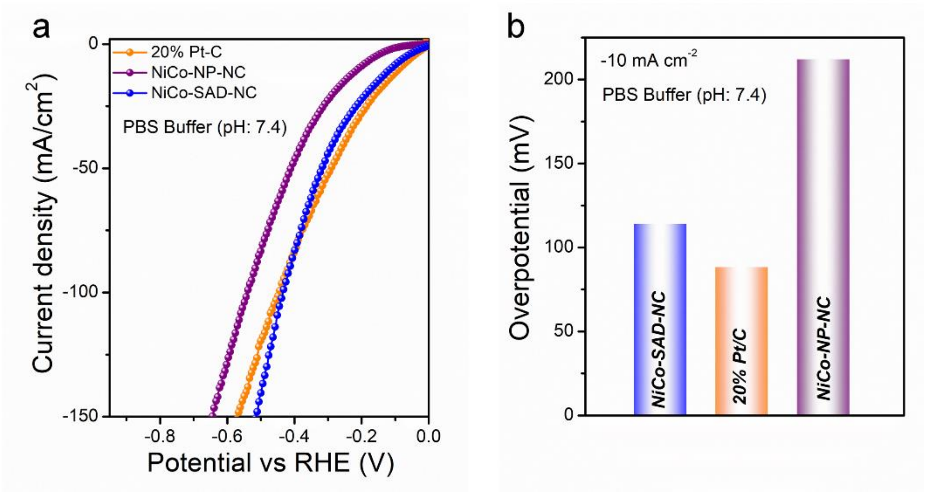

Supplementary Figure 30 | HER performance in buffer media. a, and **b**, HER LSV polarization curve and the overpotentials required to reach -10 mA cm^{-2}, respectively, in PBS solution (pH = 7.4).

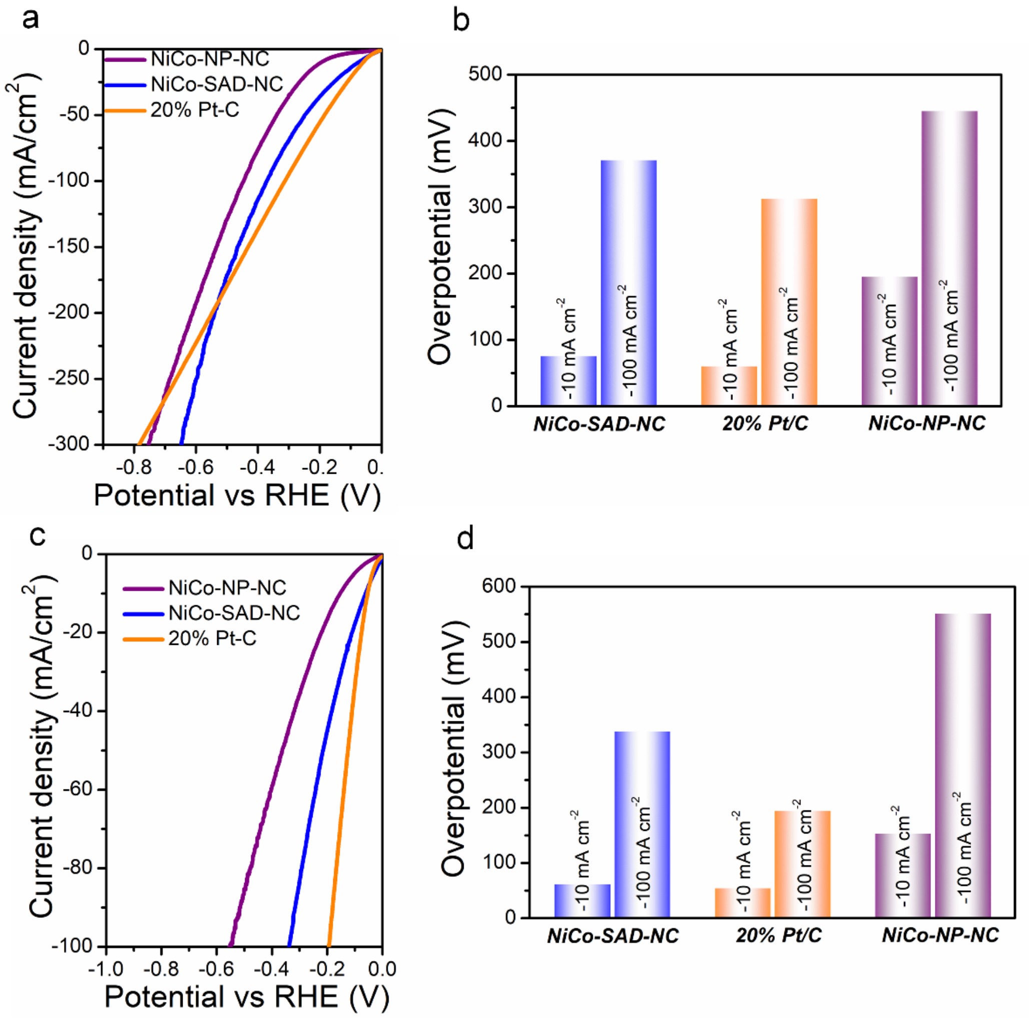

Supplementary Figure 31 | HER performance on CFP substrate. a, and **b**, HER LSV polarization curve and the overpotentials required to reach -10 mA cm^{-2}, respectively, on carbon fiber paper (CFP) current collector in 1 M KOH. **c,** and **d**, HER LSV polarization curve and the overpotentials required to reach -10 mA cm^{-2}, respectively, on CFP current collector in 0.5 M H_2SO_4.

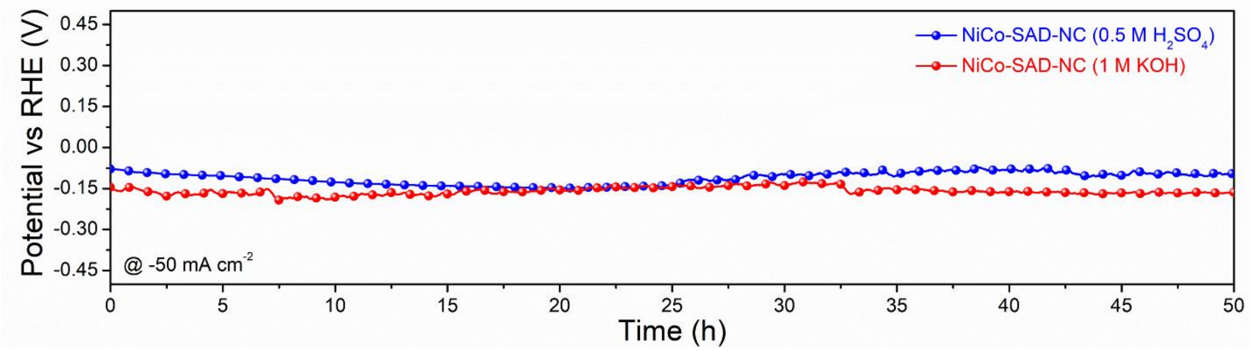

Supplementary Figure 32 | Long-term chronopotentiometric stability test for NiCo-SAD-NC in 0.5 M H_2SO_4 and 1 M KOH at a current density of -50 mA cm^{-2}.

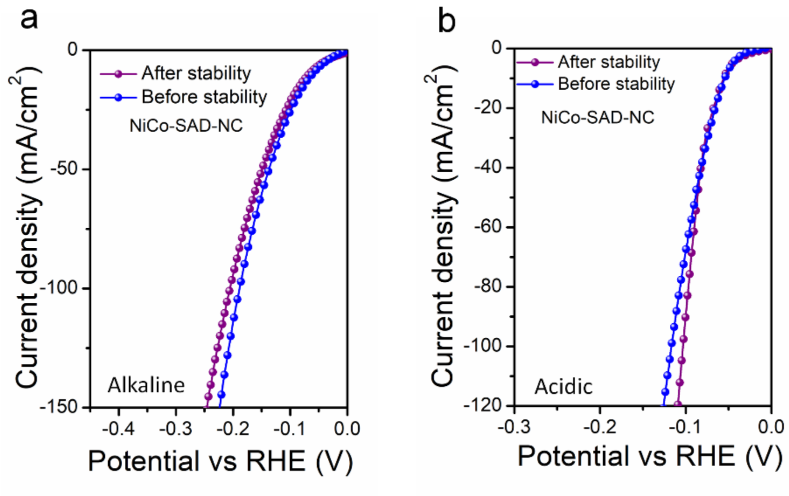

Supplementary Figure 33 | HER LSV curves before and after stability test. a, and **b**, HER LSV polarization curve before and after the stability test in alkaline and acidic media, respectively.

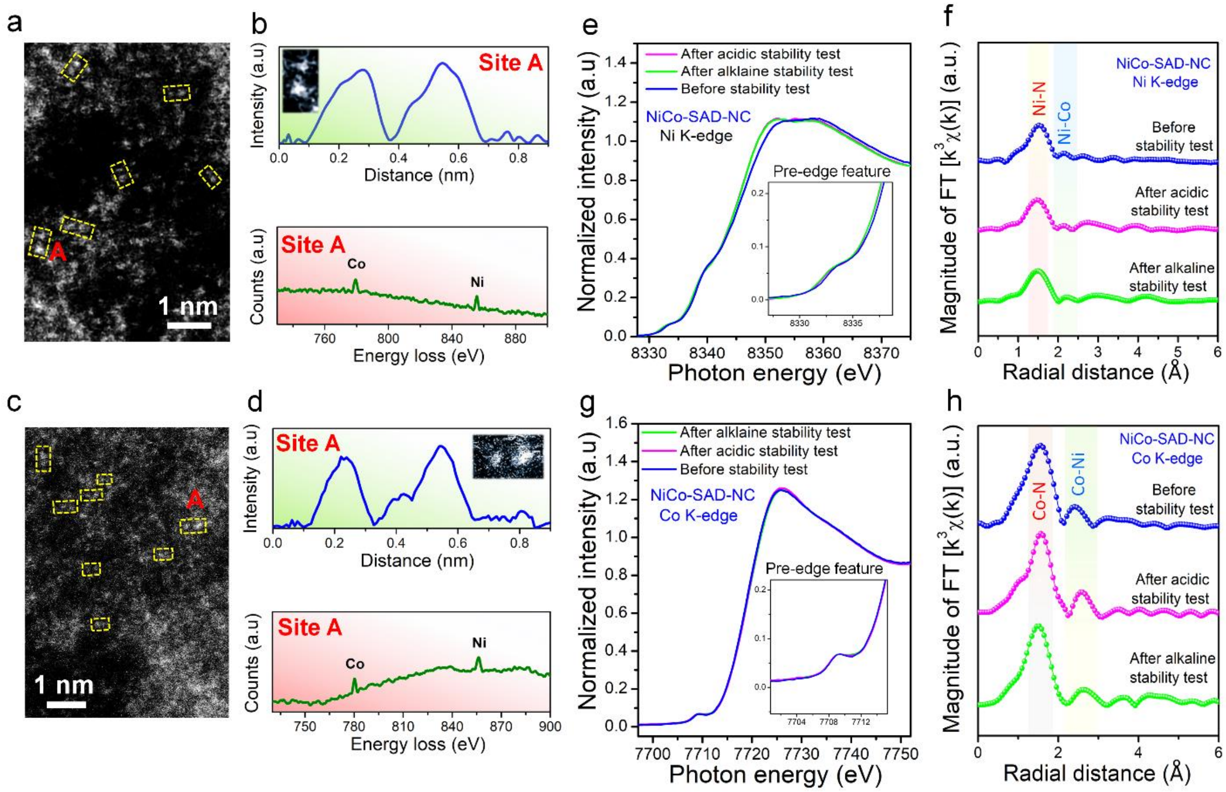

Supplementary Figure 34 | Structural characterizations after the stability test. a,**b**, Aberration-corrected HAADF-STEM image with yellow squares showing the dimer sites (**a**), and corresponding intensity profile and EEL spectrum obtained at site A (**b**) for NiCo-SAD-NC after stability test in alkaline media. **c**,**d**, Aberration-corrected HAADF-STEM image with yellow squares showing the dimer sites (**c**), and corresponding intensity profile and EEL spectrum obtained at site A (**d**) for NiCo-SAD-NC after stability test in acidic media. **e**,**f**,**g**,**h**, experimental Ni K-edge XANES (**e**), Ni K-edge FT-EXAFS (**f**), Co K-edge XANES (**g**), and Co K-edge FT-EXAFS (**h**) spectra of NiCo-SAD-NC after stability test in alkaline and acidic media.

Supplementary Fig. 34a-d showed the STEM images along with intensity profiles and EEL spectra taken at site A confirmed the existence of NiCo dimer sites after the stability test in both alkaline and acidic media. The overlapping Ni/Co K-edge XANES spectra of NiCo-SAD-NC taken before and after the alkaline/acidic stability test further revealed that the Ni/Co oxidation states along with pre-edge features remained intact (Supplementary Fig. 34e,g). Similarly, the Ni/Co K-edge FT-EXAFS spectra confirmed that the Ni/Co-N and Ni-Co bonding in the NiCo-SAD-NC also remained intact after the stability test (Supplementary Fig. 34f,h).

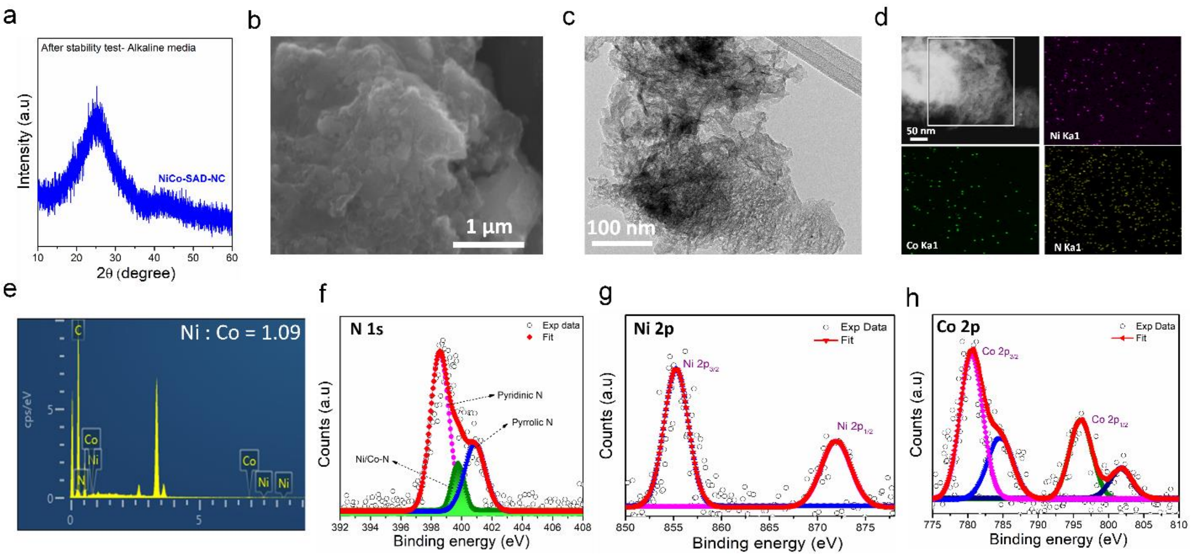

Supplementary Figure 35 | Structural characterizations after the alkaline stability test. a, **b**, **c**, **d**, and **e**, XRD pattern, FESEM image, TEM image, HAADF-STEM images and corresponding EDS elemental mapping, and EDS pattern, respectively, of NiCo-SAD-NC after stability test in alkaline media. **f**, **g**, and **h** Fitted deconvoluted N *1s*, Ni *2p* and Co *2p*, respectively, XPS spectra of NiCo-SAD-NC after stability test in alkaline media.

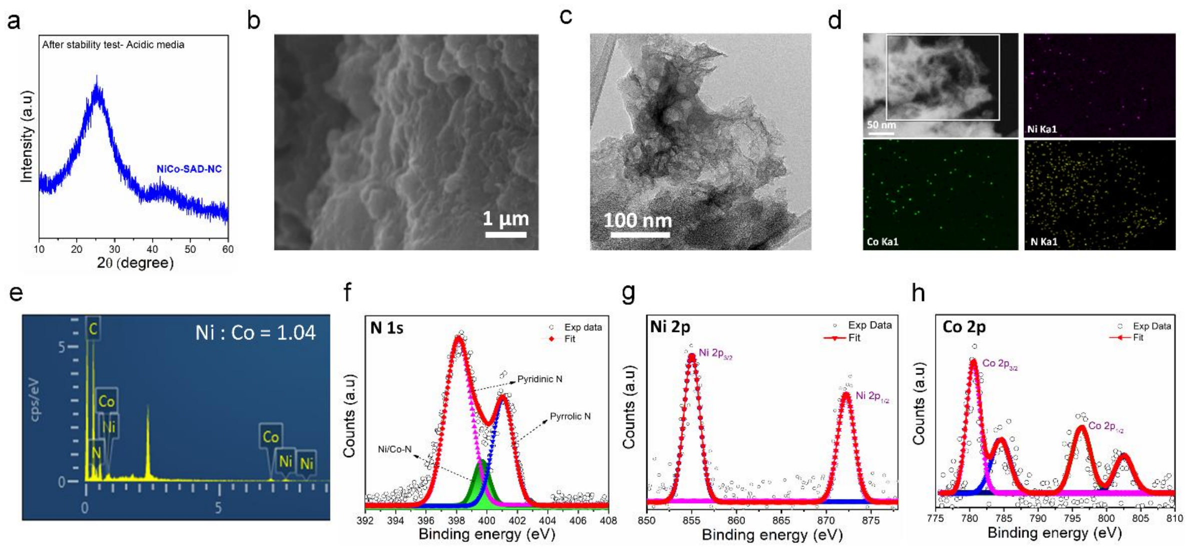

Supplementary Figure 36 | Structural characterizations after the acidic stability test. a, **b**, **c**, **d**, and **e**, XRD pattern, FESEM image, TEM image, HAADF-STEM images and corresponding EDS elemental mapping, and EDS pattern, respectively, of NiCo-SAD-NC after stability test in acidic media. **f**, **g**, and **h** Fitted deconvoluted N *1s*, Ni *2p* and Co *2p*, respectively, XPS spectra of NiCo-SAD-NC after stability test in acidic media.

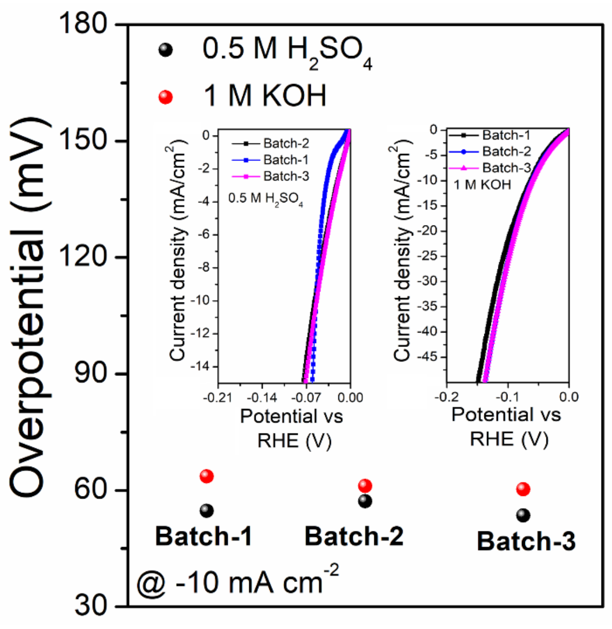

Supplementary Figure 37 | Reproducibility test of the NiCo-SAD-NC for HER in acidic and alkaline media.

Supplementary Table 6. Comparison table of the HER performance of NiCo-SAD-NC with reported HER electrocatalysts.

**Catalyst**	**η@-10 mA/cm² in acidic media (mV)**	**η@-10 mA/cm² in alkaline media (mV)**	**Reference**
**Comparison with single atom catalyst**			
NiCo-SAD-NC	54.7	61	**This work**
Pt-Ru dimer	50		*Nat. Commun.* **10**, 4936 (2019)
Pt-SA/S–C	53		*Nat. Commun.* **10**, 4977 (2019)
Ru@Co-SAs/N-C	57	7	*Nano Energy* **59**, 472-480 (2019)
Pt_1/N-C	19	46	*Nat. Commun.* **11**, 1029 (2020)
W-CoP	48	40	*ACS Sustainable Chem. Eng.* **8**, 14825-14832 (2020)
Pt/np-$Co_{0.85}Se$	58	58	*Nat. Commun.* **10**, 1743 (2019)
Fe/GD	66		*Nat. Commun.* **9**, 1460 (2018)
CoN_4-SAC		111	*Adv. Funct. Mater.* **31,** 2100547 (2021)
$Ru_{SA}CoFe_2$/G		164	*Energy Environ. Sci.*, **13**, 5152-5164 (2020)
Co_1/PCN		89	*Nat. Catal.* **2**, 134–141 (2019)
Co-SA@NCA		78.6	*Chem. Eng. J.*, **410**, 128359 (2021)
Pt@PCM	105	139	*Sci. Adv.* **4**, eaao6657 (2018)

Mo-SAC	154	132	*Angew. Chem. Int. Ed.* **56**, 16086 –16090 (2017)
Mo SAs/ML-MoS_2	107	209	*ACS Nano* **14**, 767–776 (2020)
Pt-SA decorated VS_2	77		*ACS Nano* **14**, 5600-5608 (2020)
Ni NP|Ni-N-C		147	*Energy Environ. Sci.* **12**, 149-156 (2019)
Co-C-N	138	178	*J. Am. Chem. Soc.* **137**, 15070−15073 (2015)
Co-NG-MW film	127		*Adv. Mater.* **30**, 1802146 (2018)
Ni_{SA}-MoS_2	110	98	*Nano Energy* **53**, 458-467 (2018)
Co-SAS/HOPNC	137		*Proc. Natl. Acad. Sci. U.S.A.* **115**, 12692−12697 (2018)
**Comparison with nanomaterials-based catalyst**			
Pt_3Ni_2 NWs-S/C	70		*Nat. Commun.* **8**, 14580 (2017)
Pt-Ni nanowires		13	*Adv. Mater.* **31**, 1807780 (2019)
SAP-Mo_2C-CS	36		*Angew. Chem. Int. Ed.*, **59**, 23791-23799 (2020)
PtNi-O/C	70		*J. Am. Chem. Soc.* **140**, 9046−9050 (2018)
Mn-doped FeP/$Co_3(PO_4)_2$	27	85	*ChemSusChem* **12**, 1334–1341 (2019)
3D-NiCoP	80	105	*Nano Res.* **12**, 375–380 (2019)
CoNi@NC	142		*Angew. Chem. Int. Ed.*, **54**, 2100-2104 (2015)
CP/CTs/Co-S		190	*ACS Nano* **10**, 2342-2348 (2016)

Co-Ni-P-300	150	*Chem. Commun.* **52**, 1633-1636 (2016)
Co-NiS_2 NSs	80	*Angew. Chem. Int. Ed.* **58**,18676 –18682 (2019)
NiCoN/C	103	*J. Am. Chem. Soc.* **140**, 610-617 (2018)
$NiCo_2S_4$	65	*Nano Energy* **24**, 139-147 (2016)
NiO/Ni/CNT	86	*Nat Commun.* **5**, 4695 (2014)
